# Atomic‐Level Engineering of Synthetic Receptors for Enhanced Virus Detection and Removal

**DOI:** 10.1002/adhm.202502043

**Published:** 2025-08-25

**Authors:** Eda Akin, Ekin Sehit, Nastasia Sanda Moldovean‐Cioroianu, Sahana Tavaragondi, Sophia Slenczka, Roderich Süssmuth, Friedrich Jurk, Manuel van Gemmeren, Zeynep Altintas

**Affiliations:** ^1^ Institute of Materials Science Kiel University 24143 Kiel Germany; ^2^ Institute of Chemistry Technical University of Berlin 10623 Berlin Germany; ^3^ Otto Diels‐Institute of Organic Chemistry Kiel University 24118 Kiel Germany; ^4^ Kiel Nano, Surface and Interface Science (KiNSIS) Kiel University 24118 Kiel Germany

**Keywords:** epitope‐specific affinity materials, imprinted materials, in‐silico virus receptor design, molecular dynamics, QCM sensor, virus detection, virus‐selective membrane filtration

## Abstract

Virus sensing and removal are critical for public health, particularly in preventing the spread of infectious diseases and ensuring safe water, air, and clinical environments. Current virus detection tools utilize recognition elements suffering from high cost, low stability, and specificity, and time‐consuming production methods. Meanwhile, conventional virus removal techniques are often hindered by inefficiency, complexity, and the potential for harmful byproducts. Herein, this study successfully addressed these challenges by employing extensive computational techniques to design and optimize epitope‐specific novel artificial ligands for virus detection and removal, utilized in two major applications: biosensing and membrane filtration. Virus‐specific, computationally designed imprinted receptors (CIRs) functionalized on quartz crystal microbalance (QCM) platforms, developed in this work, allow human pathogenic virus detection with high sensitivity (limit of detection = 0.064 fM) in complex media such as tap water and human serum, while providing high selectivity and specificity. Moreover, CIR integrated polyvinylidene fluoride (PVDF) and polyethersulfone (PES) membranes resulted in an efficient virus removal from contaminated water with a 100% purification rate. This synergistic approach highlights the potential of computationally derived imprinted synthetic ligands in advancing point‐of‐care diagnostics and water treatment technologies for virus sensing and removal.

## Graphical abstract

Advanced computational techniques are employed to design and optimize computationally designed imprinted receptors (CIRs) for virus detection and removal. CIR‐conjugated piezoelectric sensor achieves highly sensitive virus detection in water and human serum. CIR‐integrated membranes remove 100% of pathogenic viruses from contaminated water. The interdisciplinary approach, combining computational modeling with experimental validation, provides a proof‐of‐concept for point‐of‐care diagnostics and water treatment technologies.

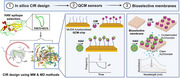

## 1 Introduction

The increasing prevalence of viral infections and contamination of environmental sources with pathogenic viruses pose significant challenges to global health and economy. Beyond the immediate health toll, these viral outbreaks lead to substantial economic losses.^[^ ^1^ ^]^ In such scenarios, the early detection and containment of viruses is critical to mitigating their far‐reaching impacts. Thus, effective virus diagnostic tools and removal technologies are essential for addressing the challenges posed by viral infections and contamination. Recent advances in point‐of‐care virus diagnostics include plasmonically active lateral flow immunoassay,^[^ ^2^ ^]^ field effect transistors,^[^ ^3^, ^4^ ^]^ multiplexed nanopore sensor,^[^ ^5^ ^]^ real‐time multimeric aptamer assay,^[^ ^6^ ^]^ plasmonic nanostructures,^[^ ^7^ ^]^ and electrochemical aptasensors.^[^ ^8^ ^]^ Although these platforms provide sensitive virus recognition, most of them utilize proteinaceous natural receptors (e.g., antibody, enzyme) which are costly and prone to denaturation. On the other hand, synthetic affinity elements such as aptamers require a tedious and expensive optimization and production process. Furthermore, currently utilized wastewater treatment methods are insufficient to inactivate majority of viruses. Despite the utilization of micro‐filtration and ultra‐filtration membranes for elimination of biological entities, the effectiveness of these membranes as hygienic barriers is still a concern due to their submicron pore size.^[^ ^9^ ^]^ Current strategies to address this issue focus on modification of membrane pores with hydrophilic polymers such as graft‐polymerized zwitterionic SPP ([3‐(methacryloylamino) propyl] dimethyl (3‐sulfopropyl) ammonium hydroxide)^[^ ^10^ ^]^ and polyethyleneimine (PEI) crosslinked with terephthalaldehyde (TA).^[^ ^11^ ^]^ Even though the functionalization of membranes significantly increased virus removal, the modified membranes were not entirely effective in eliminating all viral pathogens.

Polymeric imprinted binders are cost‐effective and stable alternatives to natural ligands.^[^ ^12^ ^]^ However, imprinting large biomolecules such as proteins, viruses, bacteria is challenging due to the high costs of obtaining large quantities of whole biomolecules, as well as their structural complexity, intricate dynamic behavior and low stability under polymerization conditions. Furthermore, the binding sites resulted from large templates exhibit reduced selectivity making it difficult to achieve precise imprints.^[^ ^13^ ^]^ Therefore, epitope‐mediated receptors, which are tailored for a specific fragment of the analyte (i.e., the epitope) rather than the whole structure, offer a more efficient approach for targeting large biostructures.

In addition, the trial‐and‐error‐based experimental studies are commonly performed to optimize synthetic receptor composition.^[^ ^14^ ^]^ On the other hand, the traditional time‐consuming and resource‐intensive empirical receptor design approach can be replaced by molecular docking and molecular dynamics (MD) techniques.^[^ ^15^, ^16^ ^]^ These methods offer unparalleled precision in predicting molecular interactions, enabling researchers to swiftly model and optimize binding affinities, interaction profiles, and structural conformations of key components, such as template molecules, functional monomers, and cross‐linkers, before synthesis.

Here, these challenges including the high cost, structural complexity, and reduced selectivity — were addressed through a holistic approach starting from rational design of synthetic virus ligands to implementing them in advanced applications, including biosensing and bioselective membrane development, with Hepatitis A virus (HAV) serving as the model analyte. In order to achieve this, comprehensive MD studies were performed for the meticulous screening of potential HAV epitopes, being among the most challenging biological compounds. Additional molecular docking and MD studies were employed for the selection of functional monomers and cross‐linkers, along with their optimal ratio, to compose the synthetic binders. This in‐silico design strategy enabled the efficient identification of the optimal composition for HAV‐specific epitope‐mediated ligands, significantly reducing the trial‐and‐error based time‐intensive optimization studies and accelerating development. By leveraging extensive computational methods, two epitope‐specific computationally–designed imprinted receptors (i.e., CIR‐1 and CIR‐2) were developed, in a precise, efficient, and cost‐effective manner, while enhancing the binding performance and selectivity toward HAV. CIRs were produced using virtually optimized polymer compositions via solid‐phase synthesis^[^ ^17^ ^]^ and further characterized with diverse methods including dynamic and electrophoretic light scattering, FT‐IR, Raman, and XPS spectroscopies. Additionally, the CIRs were applied as capturing agents for sensitive recognition and selective removal of HAV in various media. Two quartz crystal microbalance (QCM) based sensing platforms were developed using CIR‐1 and CIR‐2 as HAV specific recognition elements for mass‐sensitive HAV detection. Moreover, CIR integrated polyvinylidene fluoride (PVDF) and polyethersulfone (PES) membranes exhibited a significant capability to fully eliminate HAV from contaminated water samples. This interdisciplinary strategy integrating computational and experimental methods lays a foundation for a proof‐of‐concept which is expected to advance point‐of‐care diagnostics and water treatment technologies for virus sensing and removal with broader implications for pathogen detection, environmental monitoring, and biomedical diagnostics.

## 2 Results and Discussion

### 2.1 Computational Design of CIRs

#### 2.1.1 Template Selection and Structural Stability

**Figure 1:**
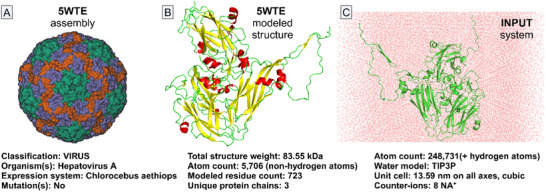
Cryo‐EM structure for Hepatitis A virus full particle A), the structure of the input solute B), and the solvated and minimized system C) subjected to 1 µs MD production using CHARMM27^[^ ^19^ ^]^ all‐atom force field and TIP3P water model.

The HAV capsid (PDB: 5WTE shown in **Figure** **1**) consists of three main viral protein (VP) subunits: VP1 (chain A), forming the outer layer and mediating host interactions; VP2 (chain B), stabilizing the capsid and inter‐subunit interactions; and VP3 (chain C), enhancing capsid stability and genome encapsulation.^[^ ^18^ ^]^ In order to identify two potential epitopes that will be used for all‐atom investigations and consequently in CIRs design, MD productions of the solvated and energy minimized HAV structure were conducted for 1 µs. The gyration radius (Rg) of the whole structure remained stable (≈5 nm), with minor fluctuations between 400–600 ns, suggesting potential structural changes. In good agreement with the root‐mean‐square deviations (RMSD) profile, solvent accessible surface area (SASA) analysis indicated initial fluctuations – prior to 200 ns – while decreasing afterwards from 360 to 315–320 nm^2^. Additionally, root‐mean‐square fluctuations (RMSF) analysis identified flexible, exposed HAV regions, with highly fluctuating residues likely in loops/external capsid parts, while low‐fluctuation residues represent core HAV structural regions.

Using the RMSF and MD trajectory visualization, two regions were identified as potential epitopes for further investigations: ASN235‐TYR242 (epitope 1 at the C‐terminal domain) and THR70‐HIS76 (epitope 2 at the N‐terminal domain). Their higher atomic fluctuations (up to 0.4 nm) indicate moderate‐to‐high flexibility and surface exposure, essential for strong molecular interactions.

The structural stability of the epitopes was assessed via secondary structure analysis (SSA) using the Timeline tool from VMD.^[^ ^20^ ^]^ The secondary structural components of both potential epitopes mainly consist of turns and extended configurations which link two β‐sheets. Importantly, SSA revealed structural transitions in epitope 1 from turn to extended configurations, alongside multiple isolated bridge structures. The later configurations (isolated bridges) are typically less stable, due to their nature to form and break within very short time frames. In contrast, HAV epitope 2 demonstrated great structural stability over time, without any structural transitions occurring during the 1 µs MD production time.

#### 2.1.2 Molecular Docking

To conduct a preliminary screening of functional monomers (FMs), the binding affinities of 18 FMs docked against the HAV epitope (epitope 2) were investigated. It is worth noting that docking studies and analyses were conducted for both epitopes; however, based on the findings in Section 2.1.1, the core focus will be exclusively on the results pertaining to epitope 2.

The statistical analysis of molecular docking data shows that, on average, epitope 2 exhibits stronger overall binding energy (E_binding_: −61.619 kcal mol^−1^) compared to epitope 1 (E_binding_: −51.785 kcal mol^−1^), primarily due to more substantial electrostatic interactions (epitope 2′s E_electrostatic_: −60.388 kcal mol^−1^ versus epitope 1′s E_electrostatic_: −48.139 kcal mol^−1^). The mean value of van der Waals (vdW) interactions is slightly higher for epitope 1 (E_vdW_: −9.343 kcal mol^−1^) compared to epitope 2 (E_vdW_: −6.294 kcal mol^−1^). The energy components obtained per each FM docked against each HAV epitope are illustrated in Table S1 (Supporting Information). Correlation analysis was performed to further evaluate the dependencies between energy components, suggesting that changes in vdW interactions yielded a greater impact, particularly for epitope 2, on the overall energy and binding affinity of the FMs (Figure S1, Supporting Information).

For epitope 2, BAP, HEMA, and NOBE monomers exhibited strong vdW forces and good binding affinities. BAP's pyridine ring enables π‐π stacking, while its amide groups form H‐bonds, enhancing vdW and electrostatic interactions. Despite neutrality, both HEMA and NOBE engage in electrostatic interactions through induced dipoles and H‐bonds. DAM and DEM monomers, due to their size and charge, also showed promising binding affinities.

With respect to the overall characteristics of the two epitopes, epitope 1 is more hydrophilic, while epitope 2 also includes hydrophobic regions. Consequently, as confirmed by the structural analysis of both HAV epitopes (Section 2.1.1), the obtained docking results, and that epitope 2 owns a great potential to interact with both hydrophobic and hydrophilic monomers – epitope 2 was selected as the template molecule for all subsequent investigations.

#### 2.1.3 All‐Atom Molecular Dynamics

In order to further refine and validate the docked template‐functional monomer (T‐FM) complexes, extensive all‐atom MD productions were performed for 100 ns per run, in multiple replicas, for each T‐FM combination used in molecular docking studies (Table S1, Supporting Information). While molecular docking provides a static binding approximation using scoring functions, MD simulations offer a dynamic view, assessing complex stability, solvent effects, and interaction energies. MD trajectories also reveal alternative T‐FM binding modes, refining and validating FM selection.

Initially, MD simulations for all FMs were performed in 1:1 T‐FM ratio. The number of atomic contacts within 0.6 nm atom distances was analyzed, considering H‐bonds (0.27–0.35 nm), vdW forces (0.3–0.4 nm), and electrostatic interactions (>0.4 nm). Accordingly, epitope 2 exhibited the highest close‐range contacts with acidic monomers, particularly AMPSA and MEP, followed by ITA and PVA. For the basic FMs, contact numbers were consistent across monomers, peaking at ≈600 contacts with PAS and BAP, followed by DAM and DEM. These trends reflected the role of vdW forces and H‐bonds, and correlated with the highest binding affinities discussed in Section 2.1.2.

**Figure 2:**
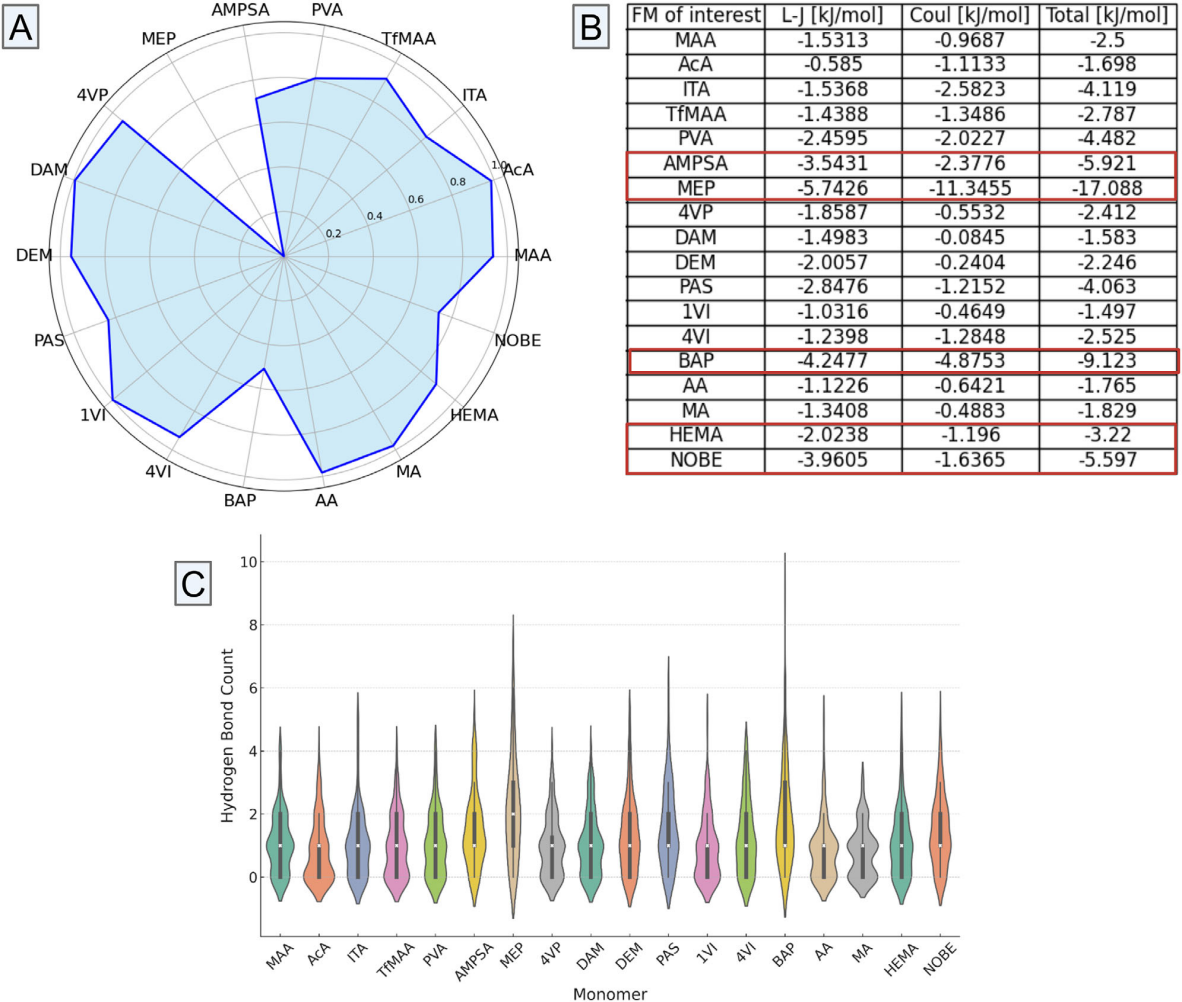
The average total interaction energy analysis between epitope 2 and FMs in 1:1 ratio A), comprising both L–J and Coulomb contributions (in kJ mol^−1^) B). The average H‐bond counts between epitope 2 and FMs for atom pairs within 0.35 nm, in 1:1 ratio, are represented in sub‐figure C). The median value (50th percentile) of the data distribution is represented by the white dots, and the thick bars indicate the interquartile ranges. * MAA – Methacrylic acid; AcA – Acrylic acid; ITA – Itaconic acid; TfMAA – 2‐(Trifluoromethyl)acrylic acid; PVA – p‐Vinyl benzoic acid; AMPSA – 2‐Acrylamido‐2‐methyl‐1‐propane sulfonic acid; MEP – 2‐(Methacryloyloxy)ethyl phosphate; 4VP – 4‐Vinylpyrrolidone; DAM – 2‐(Dimethylamino)ethyl methacrylate; DEM – 2‐(Diethylamino)ethyl methacrylate; PAS – p‐Aminostyrene; 1VI – 1‐Vinylimidazole; 4VI – 4‐Vinylimidazole; BAP – 2,6‐Bis(acrylamido)pyridine; AA – Acrylamide; MA – Methacrylamide; HEMA – 2‐Hydroxyethyl methacrylate; NOBE – N,O‐bis methacryloyl ethanolamine; L‐J – Lennard‐Jones; Coul – Coulomb.

The H‐bond analysis (**Figure** **2**) offered additional insights into the interaction mechanism between the template and monomers, confirming that AMPSA, MEP, PAS, as well as BAP, NOBE, and HEMA, were highly involved in strong H‐bond interactions with epitope 2. Interestingly, despite their lower (direct) contact numbers within 0.6 nm, AcA, ITA, and MA monomers still showed significant H‐bond patterns with the template molecule. This may be due to (i) small, flexible monomers aligning their functional groups for directional H‐bonds, (ii) strong functional moieties enabling long‐range donor‐acceptor interactions, and (iii) solvent bridges mediating H‐bonds formation. Nevertheless, the highest H‐bond contributions were noted for AMPSA and MEP (acidic FMs), PAS and BAP (basic FMs), alongside NOBE and HEMA (neutral FMs). Among these FMs, the strength of H‐bond interactions follows the ranking: MEP > BAP > PAS > NOBE > AMPSA > HEMA.

The total interaction energy (Figure 2A,B) sums Lennard–Jones (L–J) and Coulomb potentials, capturing vdW, repulsions, and electrostatics. Both components reflect the balance of attractive and repulsive forces. H‐bonds, being highly directional, were analyzed separately, as L–J and Coulomb terms alone cannot fully capture them.

Among acidic FMs, MEP showed the strongest interaction (−17.088 kJ mol^−1^), driven by its highly negative Coulomb potential (−11.345 kJ mol^−1^), indicating electrostatic dominance. AMPSA (with total energy of −5.921 kJ mol^−1^) indicates balanced L‐J and Coulomb contributions. PVA and ITA interacted mainly via Coulomb forces, while AcA had the weakest interactions, suggesting insufficient vdW and electrostatic stability. Among basic FMs, BAP had the strongest interaction energy (−9.123 kJ mol^−1^) via balanced electrostatic and vdW forces. PAS showed moderate kinetics, while 4VP and DAM had significantly weaker interactions with lower Coulomb and L‐J contributions. Lastly, among the neutral FMs, NOBE stood out with its strongest total interaction energy (−5.597 kJ mol^−1^), followed by HEMA (−3.302 kJ mol^−1^), while AA and MA had weaker energy profiles. The NOBE's and HEMA's hydrophobic regions and large sizes enhanced vdW forces, with flexible conformations maximizing epitope contact. As a result of the 1:1 T‐FM ratio investigations, the strongest FM candidates – AMPSA (acidic), MEP (acidic), and BAP (basic) – were used for further MD studies. Additionally, NOBE and HEMA were selected as the neutral counterparts in the polymerization mixtures. Based on these findings (Figure 2B), all the other FMs were excluded from the subsequent all‐atom investigations. In the following section, the all‐atom trajectories of two paired systems were analyzed: (i) epitope 2 interacting with AMPSA‐BAP‐HEMA in 1:5 and 1:7 ratios, and (ii) epitope 2 in interaction with MEP‐BAP‐NOBE in 1:5 and 1:7 ratios. The 1:5 and 1:7 ratios imply that the modeled systems consist of one epitope molecule and either 5 or 7 molecules of each FM (e.g., 5 molecules of AMPSA, 5 molecules of BAP, and 5 molecules of HEMA).

**Figure 3:**
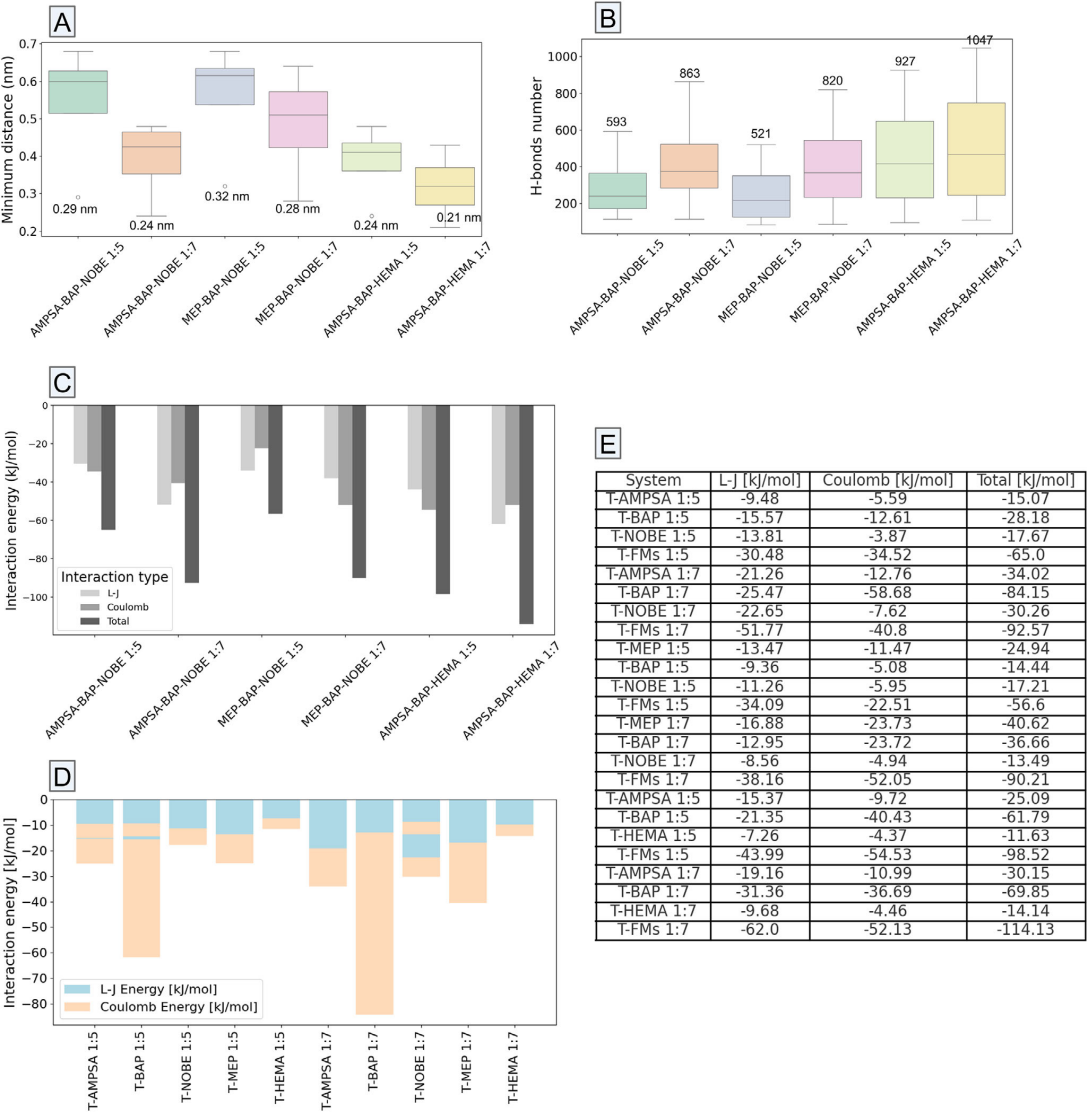
Boxplots of minimum distances A) and total number of H‐bonds B) across systems at 1:5 versus 1:7 ratios. Bar charts of the average total interaction energies for T‐FMs complexes C), and for the individual FM components in both 1:5 and 1:7 ratios D). Lennard‐Jones and Coulomb contributions, alongside their summations, are indicated in the table (E). * AMPSA – 2‐Acrylamido‐2‐methyl‐1‐propane sulfonic acid; BAP – 2,6‐Bis(acrylamido)pyridine; NOBE – N,O‐bis methacryloyl ethanolamine; MEP – 2‐(Methacryloyloxy)ethyl phosphate; HEMA – 2‐Hydroxyethyl methacrylate; L‐J – Lennard‐Jones.

**Figure 4:**
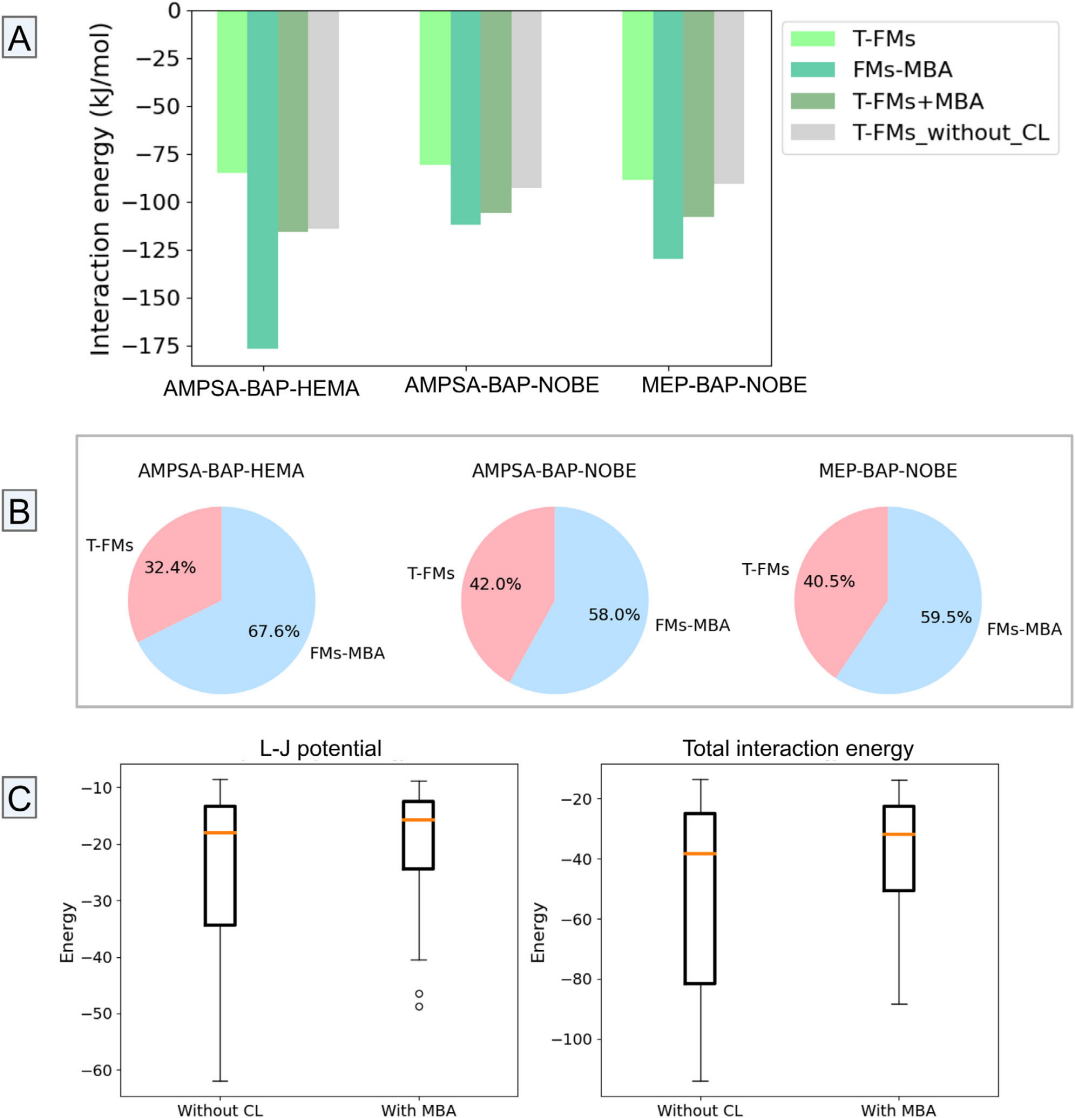
Cross‐linking analysis using MBA for the good‐/best‐performing CIR systems in 1:7 ratio A), and the pie charts B) for a visual breakdown of the contribution (%) of T‐FMs versus FMs‐MBA interactions. The boxplot analysis of the impact of CLs’ addition on both L–J contributions and total interaction energies (kJ mol^−1^) across the best‐performing CIR systems (in 1:7 ratio) C). * MBA – N,N´‐methylenebisacrylamide; AMPSA – 2‐Acrylamido‐2‐methyl‐1‐propane sulfonic acid; BAP – 2,6‐Bis(acrylamido)pyridine; HEMA – 2‐Hydroxyethyl methacrylate; NOBE – N,O‐bis methacryloyl ethanolamine; MEP – 2‐(Methacryloyloxy)ethyl phosphate; L‐J – Lennard‐Jones; T‐FMs – template‐functional monomers; FMs‐ functional monomers; CL – cross‐linker.

The AMPSA‐BAP‐NOBE complex was analyzed for epitope 2 interactions but excluded from top‐performing systems after thorough evaluation (**Figures** **3** and **4**). This (1:5) system had a 0.29 nm median T‐FMs distance, while HEMA's addition (1:7) in AMPSA‐based systems improved interatomic distances (of 0.21 nm) and interaction energy, proving HEMA monomer more effective than NOBE. Moreover, an overall competing behavior was observed between AMPSA‐BAP‐NOBE (T‐FMs total interaction energy of −92.57 kJ mol^−1^) and MEP‐BAP‐NOBE (T‐FMs total interaction energy of −90.21 kJ mol^−1^) formulations. However, MEP monomer exhibited stronger interaction patterns with the selected epitope (−40.62 kJ mol^−1^), as compared to AMPSA (−34.02 kJ mol^−1^). In this context, the MEP‐BAP‐NOBE system was selected for further investigations, alongside the AMPSA‐BAP‐HEMA formulation.

The AMPSA‐based system exhibits a strong correlation between FM ratios and H‐bond formation, enhancing CIR stability (Figure 3B). While MEP‐BAP‐NOBE (1:7) increases H‐bonds, its effect is weaker than AMPSA‐based systems, suggesting reliance on vdW and electrostatic forces coming mainly from MEP (Figure 3E). The HEMA monomer's addition (1:7) further boosts AMPSA‐BAP H‐bond formation (1047 total H‐bonds). BAP, common in both formulations, performs best in the presence of AMPSA and HEMA, peaking at 647 H‐bonds (1:7) and at 0.29 nm average minimum T‐BAP distance.

To summarize, MEP‐BAP‐NOBE and AMPSA‐BAP‐HEMA systems, particularly the latter system (Figure 3A), showcased a strong correlation between the increase in monomer ratio (1:7), decrease in template‐monomer minimum distances, and the elevated total number of H‐bonds (Figure 3B) formed with the template molecule.

Coulomb interactions dominated CIRs stability (Figure 3C–E), with BAP playing a major role in achieving large negative total energy values. HEMA enhances T‐FMs complexation, contributing the strongest energies of −98.52 and −114.13 kJ mol^−1^ in 1:5 and 1:7 ratios, respectively. While HEMA and NOBE generally have weaker contributions, they further stabilize T‐FMs systems through moderate L–J and Coulomb interactions. In the MEP‐BAP‐NOBE system, the total interaction energies are less negative but still significant (e.g., −56.60 kJ mol^−1^ in 1:5 ratio). Notably, BAP performs better in AMPSA‐based formulations, achieving −69.85 kJ mol^−1^. Overall, the top‐performing systems are AMPSA‐BAP‐HEMA (1:7, −114.13 kJ mol^−1^) and MEP‐BAP‐NOBE (1:7, −90.21 kJ mol^−1^).

#### 2.1.4 Cross‐Linking Effects

Cross‐linkers (CLs) enhance CIR design by providing additional structural integrity and stability to the polymer matrix.^[^ ^21^ ^]^ While the best‐performing CIR formulations already exhibit cross‐linking properties due to the presence of bifunctional FMs such as BAP and NOBE (Figure 4), two additional CLs, ethylene glycol dimethacrylate (EGDMA) and N,N´‐methylenebisacrylamide (MBA), were further tested in MEP‐BAP‐NOBE (1:7) and AMPSA‐BAP‐HEMA (1:7) systems. MBA only slightly improved T‐FMs interaction energies, while EGDMA weakened them by interacting more strongly with the monomers.

The cross‐linking study has surfaced several pertinent considerations and questions: (i) are additional CLs always beneficial for CIR stability? (ii) do strong FM‐CL interactions weaken T‐FM binding? (iii) could BAP/NOBE acting as CLs contribute to this detrimental effect? The results in Figure 4 suggest that, while the addition of MBA in the polymerization mixture might only slightly – if at all, improve the overall CIR stability, CLs often hinder the direct interactions between the HAV epitope and FMs. This finding suggests that whether or not CLs are beneficial, it highly depends on the specific monomers used and their intrinsic binding capabilities. Hence, the observed reduction in T‐FMs interaction strength in the presence of additional CLs may be attributed to several physical and dynamical factors: (i) restricted mobility: strong FM‐CL interactions limit FM flexibility, increasing entropic penalties and hindering optimal template binding; (ii) competitive binding: excessive FM‐CL interactions divert monomers from binding to the target analyte; and (iii) shielding effects: CLs physically block template‐monomer interactions, forming less specific binding cavities, thereby reducing rebinding efficiency. The MD trajectories obtained in this work showed that over‐crosslinking with additional CLs (e.g., MBA) reduces FMs flexibility, trapping them in less favorable energetic states for template interactions.

Two‐way ANOVA was performed for determining the actual contributions of L–J and Coulomb in both “without CL” and “with MBA” conditions. The AMPSA‐BAP‐HEMA system relied on L–J interactions, which weakened with MBA addition, while MEP‐BAP‐NOBE heavily depended on Coulomb forces, which also decreased with CLs addition. The ANOVA results (sum_sq = 13 749.65, F = 16.077, *p* = 7.065 ✗ 10^−7^) confirmed a significant effect (*p* < 0.05) of energy types across CIR systems and CL conditions. Additionally, T‐test results indicated a statistically significant difference in L–J contributions between “without CL” and “with MBA” conditions (*p* = 0.0216), with a negative t‐statistic (−2.50) indicating stronger (more negative) L–J energies without CLs. Similarly, the total interaction energies differed significantly (*p* = 0.0195), correlating strongly with L–J contributions (coefficient of 0.96). Figure 4C shows stronger L–J and total interaction energies, indicating better performance in systems without CLs. Coulomb contributions were slightly stronger without additional CLs, but not significantly (*p* = 0.0628).

Based on the computational results, the best CIR systems for targeting the selected HAV epitope 2 (seq.: TTHALFH) were AMPSA‐BAP‐HEMA (i.e., CIR‐1) and MEP‐BAP‐NOBE (i.e., CIR‐2). The total interaction energies of the selected formulations ranged between −90 and −114 kJ mol^−1^, when no additional CL molecules were included in the polymerization mixture. While the addition of CLs promoted strong FMs‐CLs interactions, the excessive cross‐linking significantly hindered T‐FMs complexation, therefore impacting the overall CIR's components kinetics. To conclude, AMPSA‐BAP‐HEMA in 1:7 ratio (i.e CIR‐1) and MEP‐BAP‐NOBE in 1:7 ratio (i.e., CIR‐2), without additional CLs, were used for synthesis of CIRs for HAV sensing and removal applications in the continuation of this work.

### 2.2 Synthesis and Characterization of CIRs

The computationally derived formulations were used for solid‐phase synthesis of CIR‐1 and CIR‐2.^[^ ^22^ ^]^ NOBE (Figure S3, Supporting Information) and BAP (Figure S5, Supporting Information) monomers were synthesized in‐house and characterized via ^1^H NMR (Figures S4 and S6, Supporting Information) and Fourier transform infrared spectroscopy (FTIR) (Figure S7, Supporting Information) to confirm their successful production.

Moreover, the HAV epitope 2 with amino‐acid sequence TTHALFH was synthesized to be used as the template and further characterized with high‐resolution mass spectroscopy and NMR techniques confirming successful production of the epitope (Figure S8, Supporting Information). HRMS (ESI) analysis showed an m/z value of 826.4202, which is in excellent agreement with the calculated value for C_38_H_55_N_11_O_10_ [M+H]⁺, 826.4206.

Both CIR‐1 and CIR‐2 were produced via solid‐phase synthesis templating rationally selected epitope 2. For this, glass beads (GBs) were utilized as solid support phase which initially went under hydroxylation and silanization steps to facilitate epitope immobilization with the aid of glutaraldehyde linking. A ninhydrin test was performed before and after epitope incubation during the solid‐phase synthesis to confirm the successful binding of the epitope to the GBs (Figure S9, Supporting Information). After anchoring the epitope template on the support, the polymerization mixtures composed of computationally selected monomers with the favorable T:FM ratios were utilized for the synthesis of artificial binders. CIR‐1 was prepared using HEMA, AMPSA and BAP, while CIR‐2 comprised of NOBE, MEP, and BAP. Furthermore, N‐(3‐aminopropyl) methacrylamide hydrochloride (APMA) was introduced in both recipes to obtain primary amine functional groups which were later utilized for covalent immobilization of synthetic ligands on sensor crystal and membrane surface. In addition, a rhodamine dye was included in the formulations to facilitate physical characterization of CIR‐conjugated surfaces. After polymerization, the GBs were washed with cold water (4 °C) to remove low‐affinity particles, short oligomers, and residual monomers. Then the high affinity CIRs were detached from the template via elution with water at 65 °C.

The hydrodynamic size and zeta potential of the synthesized imprinted nanomaterials were determined by dynamic light scattering (DLS) and electrophoretic light scattering (ELS) techniques from the eluted polymer solution. CIR‐1 depicted a smaller hydrodynamic size (246.8 ± 4.51 nm) with a polydispersity index (PDI) of 0.29 (Figure S10A, Supporting Information), while CIR‐2 exhibited a hydrodynamic size of 303.8 ± 9.58 nm (PDI: 0.35) (Figure S10C, Supporting Information). Both CIR‐1 and CIR‐2 depicted positive zeta potential values of 12.64 and 19.17 mV, respectively (Figure S10B–D, Supporting Information). Additionally, non‐imprinted polymers (NIPs) were characterized, with NIP‐1 showing a hydrodynamic size of 217.2 ± 5.97 nm (PDI: 0.30) and a zeta potential of 16.09 mV, and NIP‐2 displaying a size of 284.6 ± 22.82 nm (PDI: 0.31) and a zeta potential of 22.1 mV (Figure S11, Supporting Information). The similar size and zeta potential of NIPs to their CIR counterparts confirmed successful production.

**Figure 5:**
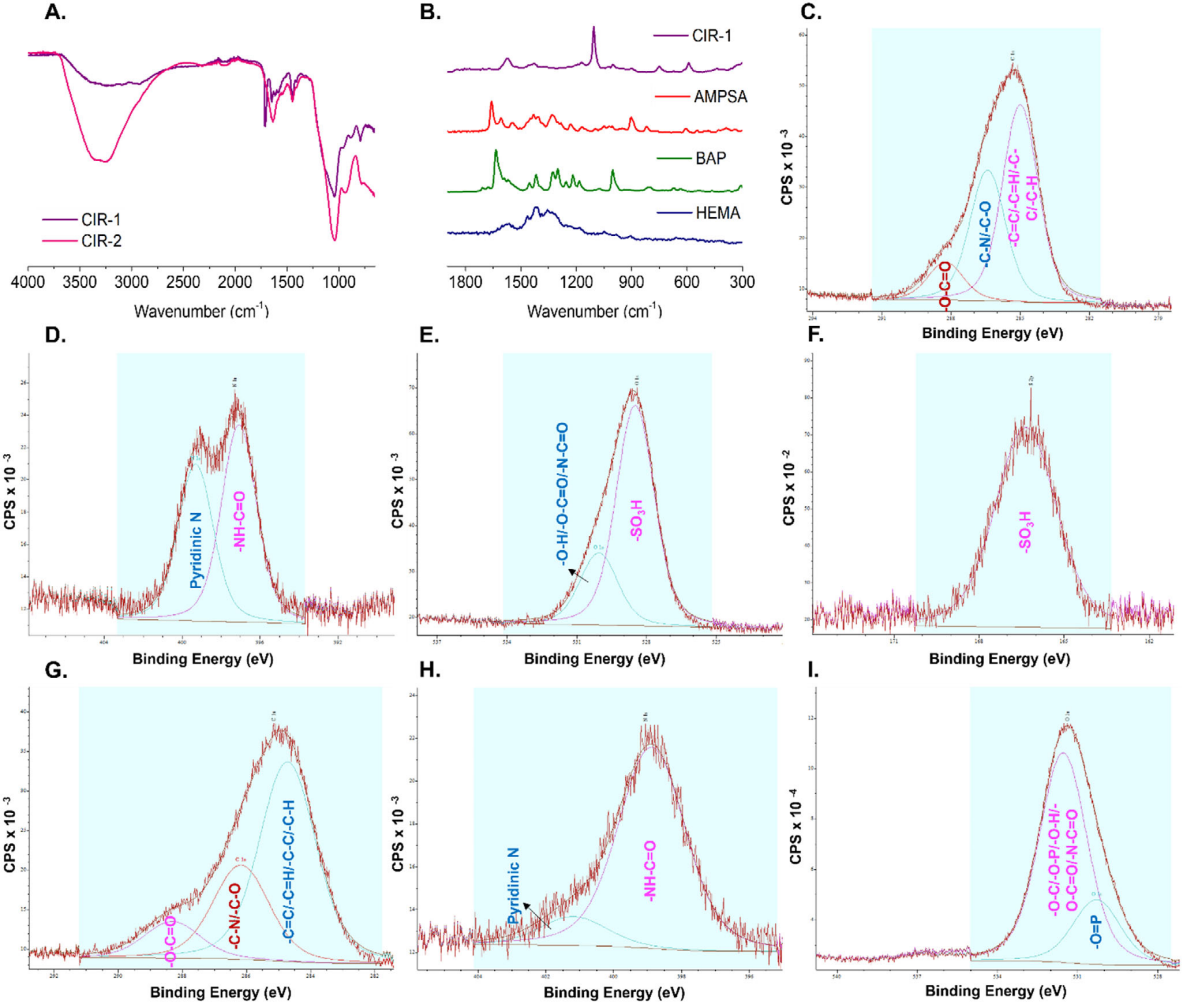
FTIR spectra of CIR‐1 and CIR‐2 A). Raman spectra of CIR‐1 and its monomers B). XPS profiles of CIR‐1 for carbon C), nitrogen D), oxygen E), and sulfur F). XPS spectra of CIR‐2 for carbon G), nitrogen H), and oxygen I).

The synthesized CIRs were characterized by FTIR to confirm their successful synthesis (**Figure** **5**A), while Raman spectroscopy was used to identify functional groups in CIR‐1 (Figure 5B) and CIR‐2 (Figure S12A, Supporting Information) by analyzing vibrational modes and investigating FMs to determine peak origins. X‐ray photoelectron spectroscopy (XPS) analysis of CIR‐1 (Figure 5C–F) and CIR‐2 (Figure 5G–I) was performed to examine the characteristic electron binding energies of their functional groups. Detailed explanation for spectroscopic analyses of CIRs is given in Supporting Information.

### 2.3 Sensor Performance

The sensing performance of computationally architectured artificial ligands was investigated using a quartz crystal microbalance (QCM‐I, Microvacuum Ltd., Hungary) as the transducing system. The resonance frequency of the piezoelectric crystal was monitored as the sensor output since this parameter is negatively proportional to the mass adsorbed on the QCM crystal.^[^ ^23^ ^]^ Herein, the alteration in the resonance frequency of the crystal was simultaneously monitored to gain insights into the sensor fabrication process and to observe the binding events between the virus and the artificial ligands.

The sensors were constructed by anchoring the CIRs on QCM chip (5 MHz, Microvacuum Ltd., Hungary) via covalent immobilization strategy. In order to achieve this, gold surface of the crystal was initially coated with self‐assembled monolayer of 11‐mercaptoundecanioc acid (MUDA) to obtain carboxylic acid functionalities which were then activated with 1‐ethyl‐3‐(3‐dimethylaminopropyl) carbodiimide (EDC) and N‐hydroxysuccinimide (NHS) mixture. The activated carboxyl groups were then utilized to couple the CIRs from their primary amine functionalities introduced by the addition of *N*‐(3‐Aminopropyl) methacrylamide (APMA) monomer during polymerization. Following the immobilization of synthetic ligands statistically, the unconjugated immobilization sites were blocked with 0.1 m ethanolamine in 10 mm phosphate buffer saline (PBS). The conjugation of CIR particles was studied with frequency readings of QCM platform as well as fluorescence imaging. Following the immobilization of synthetic ligands, a frequency decrease of 8.1 ± 1.2 and 9.7 ± 0.9 Hz was observed for CIR‐1 and CIR‐2, respectively. The slight difference in the frequency values upon conjugation on microbalance substrate could be resulted from the larger hydrodynamic size of CIR‐2 compared to CIR‐1. Furthermore, fluorescence micrographs revealed pink‐red islands upon immobilization of the rhodamine dye incorporated CIRs (Figure S14, Supporting Information). On the other hand, such fluorescent structures were not observed on MUDA coated crystal surface due to the absence of polymeric particles. Such a drastic difference in micrographs was attributed to the successful immobilization of polymeric ligands onto the sensor substrate.

**Figure 6:**
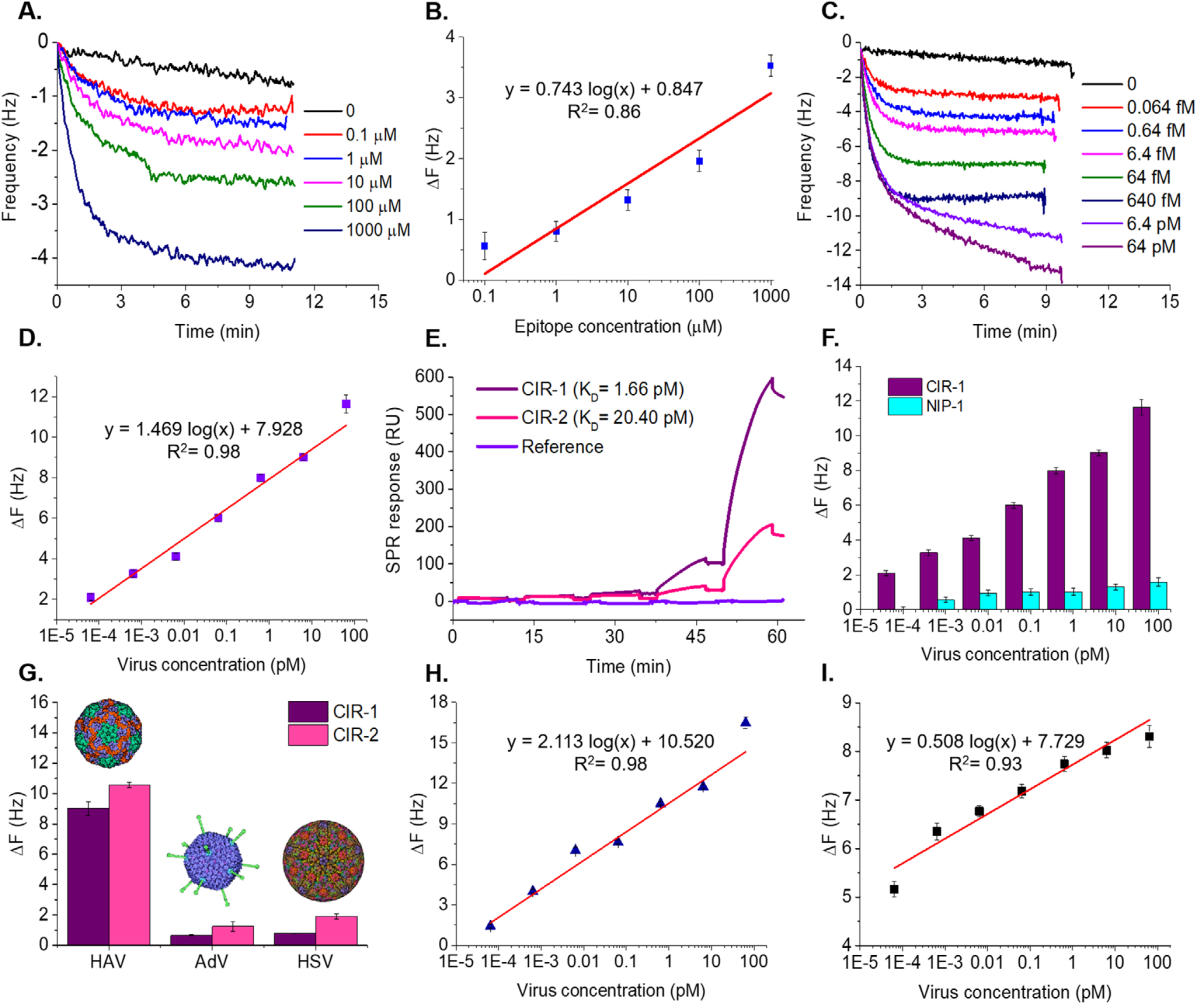
Real‐time QCM sensogram A) and logarithmic regression analysis B) of epitope detection by CIR‐1@QCM sensor. Mass‐based simultaneous HAV detection (0.064 fm – 64 pm) C) and resulting logarithmic regression analysis D) of CIR‐1@QCM sensor. SPR sensograms of HAV recognition by CIR‐1 and CIR‐2 for affinity determination E). HAV detection with computationally derived CIR‐1 compared to control non‐imprinted polymer (NIP) F). Cross‐reactivity of different pathogenic viruses (Adenovirus and Herpes simplex virus‐1) on CIR‐1 and CIR‐2 conjugated QCM sensors G). HAV detection in spiked 50% tap water H) and 1% human serum I) for a concentration range of 0.064 fm – 64 pm.

HAV epitope‐imprinted CIR‐1 and CIR‐2 were initially tested for the recognition of the epitope for a concentration range of 0.1–1000 µm in PBS buffer. Real‐time sensograms of CIR‐1 immobilized QCM (CIR‐1@QCM) were recorded upon sequential injection of the epitope solutions at different concentrations starting from of 0.1 to 1000 µm (**Figure** **6**A). The resulting sensograms demonstrated a reduced frequency readout as the epitope concentration was gradually increased. Frequency change (∆F) was calculated for each concentration with respect to reference (i.e., PBS buffer) as the sensor response. A limit of detection (LOD) of 0.1 µm for CIR‐1 was obtained with the logarithmic regression equation of ∆F (Hz) = 0.743 Log *C*_epitope_ (µm) + 0.847 with fitting value (coefficient of determination, R^2^) of 0.86 (Figure 6B). On the other hand, CIR‐2 could not detect 0.1 µm of epitope, therefore a narrower detection range between 1 and 1000 µm was studied revealing a linear correlation represented as ∆F (Hz) = 0.004 *C*_epitope_ (µm) + 0.271 with R^2^ of 0.99 (Figure S15A, Supporting Information).

As the efficient recognition of the epitope by the synthetic receptors was verified, mass‐based sensing of the target virus was studied for a wide detection range of 0.064 fm – 64 pm in PBS with 0.05% (v/v) Tween 20 (PBST). Initially, two different ligand immobilization buffers (i.e., 10 mm sodium acetate at pH 5 and 10 mm 2‐(N‐morpholino) ethanesulfonic acid (MES) pH 6) were tested for the conjugation of the polymeric receptors to improve virus binding performance. CIR‐1@QCM sensor platform prepared with sodium acetate buffer as the CIR immobilization medium yielded a larger ∆F response for HAV detection compared to MES (Figure S15B, Supporting Information). On the other hand, CIR‐2 particles exhibited better HAV detection performance when conjugated on gold surface in the presence of MES buffer (Figure S15C, Supporting Information). Therefore, in the remaining sensor studies, sodium acetate and MES buffers were used for immobilization of CIR‐1 and CIR‐2 on gold crystals, respectively. Under optimized immobilization conditions, CIR‐1 demonstrated a proportionally decreasing frequency readout as the virus concentration increased (Figure 6C). Such a reduction was due to HAV particles binding on the CIR‐1 receptors causing an increase in the adsorbed mass onto the QCM substrate. CIR‐1 successfully recognized HAV particles at concentrations as low as 0.064 fM. The logarithmic regression analysis revealed the following correlation: ∆F (Hz) = 1.469 Log *C*_HAV_ (pM) + 7.926 (R^2^ = 0.98) (Figure 6D). Similarly, CIR‐2 demonstrated an LOD of 0.064 fm with a detection range of 0.064 fm – 64 pm (Figure S15D, Supporting Information). Interestingly, the binding behavior of CIR‐2 was altered for HAV concentrations higher than 64 fm resulting in two different detection regimes, below and above 64 fm, which were represented by ∆F (Hz) = 0.1607 Ln *C*_HAV_ (pm) + 1.807 (R^2^ = 0.92) and ∆F (Hz) = 1.9869 Ln *C*_HAV_ (pm) + 6.1367 (R^2^ = 0.97), respectively.

Descriptive statistics, Kruskal‐Wallis test, as well as two‐way ANOVA were performed for statistical investigation of HAV sensing performances of the synthetic ligands. Kruskal‐Wallis test depicted no statistically significant differences between the HAV binding performances of CIR‐1 and CIR‐2 with *p*‐value of 0.1 (>0.05). In addition, two‐way ANOVA, in which virus concentration and ligand were considered as two independent variables simultaneously, depicted *p*‐value of 0.157, indicating no statistically significant difference for CIR‐1 and CIR‐2 on average. These analyses confirmed that both CIRs presented in this work can be utilized for HAV detection successfully. This agrees with the comparable total interaction energies obtained for CIR‐1 and CIR‐2 compositions in comparison to other MD recipes. On the other hand, descriptive analysis clearly demonstrated that the average sensor signal for CIR‐1 was 1.75 Hz larger than that of CIR‐2. The difference between the sensor signals of CIR‐1 and CIR‐2 was further analyzed resulting in a moderate Cohen's d size of 0.37 (between 0.2 and 0.5). This finding indicates that CIR‐1 provided enhanced sensitivity for the entire HAV concentration range which is traced to larger total interaction energy of CIR‐1, as compared to CIR‐2, obtained in our MD productions.

*In‐silico* constructed CIRs possess extraordinary durability due to their synthetic nature which allows for regeneration of the cavities for reusability. To demonstrate this, the cavities of imprinted ligands were regenerated under basic conditions, in which 50 mm NaOH was applied for 5 min after completion of the virus binding assay. Then, virus binding assay was repeated in the same manner as the initial assay to evaluate the sensing performance after regeneration. Both CIR‐1 and CIR‐2 achieved the same LOD during the initial assay (i.e., 0.064 fm) with a detection range of 0.064 fm – 64 pm of HAV (Figure S15E,F, Supporting Information). However, ∆F values recorded for HAV binding assays after regeneration were slightly lower compared to the initial assays, especially at higher HAV concentrations. This was likely due to some binding sites still being occupied by HAV from the initial assay. Nevertheless, both receptors exhibited evident reusability for HAV detection since a clear concentration‐dependent increase of ΔF was observed. The stability of CIR‐1 and CIR‐2 on sensor surfaces was investigated before and after regeneration experiments using fluorescence microscopy. The fluorescence images of the sensor surfaces did not show significant alteration after regeneration, confirming that the polymeric ligands remained intact on the crystal after basic treatment (Figure S16, Supporting Information).

Surface plasmon resonance (SPR) based ligand characterization tool (Biacore X100, Cytiva, Germany) was employed for affinity determination of CIR‐1 and CIR‐2 toward HAV (Figure 6E). Single‐cycle kinetics evaluation was performed for both ligands by fitting the experimental data into 1:1 binding model in Biacore X100 evaluation software. Dissociation constant (K_D_) was calculated as the indicator of affinity between the artificial ligands and the HAV. For the entire concentration range, CIR‐1 showed a higher SPR response than CIR‐2, which became more evident at the highest HAV concentration (i.e., 64 pm). Furthermore, CIR‐1 demonstrated 12‐fold enhanced affinity (K_D_ = 1.66 pm) than of CIR‐2 (K_D_ = 20.40 pm). The greater affinity of CIR‐1 was attributed to its enhanced kinetic behavior observed in MD simulations, thereby providing experimental confirmation of the computational analyses.

#### 2.3.1 Selectivity and Specificity of CIRs

Control binding studies were performed comparatively using non‐imprinted polymers (NIP) synthesized with the same polymer composition as of CIRs in the absence of template epitope yielding NIPs with similar size and zeta potential to their CIR counterparts. CIR‐1 demonstrated significantly larger binding response for the entire HAV detection range than of NIP‐1, which was also confirmed with two‐way ANOVA yielding *p*‐value of 1.28 × 10^−10^ (Figure 6F). Furthermore, NIP‐1 failed to recognize the lowest virus concentration (0.064 fm) and exhibited reduced sensor readout even at elevated HAV concentrations, due to the lack of selective cavities in the polymer matrix. Similarly, NIP‐2 could not detect the lowest virus concentrations, while showing concentration‐dependent binding response for the high virus concentrations (64 fm – 64 pm) (Figure S17, Supporting Information). The imprinting factor (IF) was calculated as 6.35 for CIR‐1 and 3.64 for CIR‐2, demonstrating that CIR‐1 is more selective than CIR‐2.

The binding behavior of CIRs was examined toward different non‐targeted viruses namely, Human Adenovirus‐5 (AdV) and Herpes Simplex Virus‐1 (HSV) (Figure 6G). For 6.4 pm of virus, CIR‐1 showed 13.66‐ and 11.71‐times greater signal for HAV than for AdV and HSV‐1, respectively. CIR‐2 could detect HAV with 8.44‐fold higher specificity than AdV; however, demonstrated only 5.5‐fold specificity compared to HSV. This trend was associated with the large structure of the non‐targeted HSV‐1 (155–240 nm)^[^ ^24^ ^]^ creating an elevated frequency as opposed to the HAV target with smaller size (≈27 nm).^[^ ^25^ ^]^ In such scenarios, even a small number of nonspecifically bound HSV‐1 particles could generate a larger frequency shift, compared to smaller HAV particles. Overall, both CIR‐1 and CIR‐2 exhibited much larger sensor responses for HAV compared to other viruses, further confirmed by Tukey HSD post‐hoc test revealing statistically different sensor response for HAV in comparison to AdV (*p* = 0.0035) and HSV (*p* = 0.004), proving the strong specificity of the polymeric ligands.

A pioneering virus detection platform utilizing QCM crystals with imprinted receptors was developed via a surface imprinting process resulting in successful mass‐based recognition of human rhino virus 14 (HRV 14).^[^ ^26^ ^]^ Recently, Gong and colleagues reported a colorimetric sensing platform for H5N1 detection via sandwich assay.^[^ ^27^ ^]^ H5N1 virus was imprinted on magnetic Fe_3_O_4_ core which was decorated with quantum dots (QDs). Meanwhile, H5N1‐specific aptamer was conjugated on Fe^3+^ functionalized polydopamine nanoparticles. These two nanocomposite particles were combined for H5N1 recognition achieving an LOD of 11.3 fm with an IF of 3.2. This sophisticated sensing platform provided multimodal detection of target virus at fm levels by combining multiple nanocomponents. In order to accomplish this, the authors optimized various parameters including the dosage of two different monomers, reaction time, and pH value experimentally.^[^ ^27^ ^]^ In another work, Victorious and co‐workers^[^ ^28^ ^]^ utilized a dual‐electrode electrochemical chip (DEEChip) incorporated with a barcode‐releasing electroactive aptamer for on‐site detection of porcine epidemic diarrhea virus (PEDV). The reagentless electrochemical sensor could detect clinically relevant LODs of 8 nm in spiked saliva samples. Upon aptamer library creation, five best aptamer candidates were investigated via gel‐based electrophoresis mobility shift assay. In contrast, the CIRs developed in this study exhibited enhanced sensitivity and selectivity, while significantly reducing the experimental workload required for empirical optimization. By integrating advanced computational methods, such as molecular docking and MD, we optimized receptor design within a few months, compared to the years typically required for experimental approaches. This streamlined strategy not only accelerated the development process but also enabled precise tailoring of receptor binding performance and selectivity for HAV. By minimizing trial‐and‐error experimentation, our approach improves efficiency and yields high‐performance materials suitable for practical applications.

Previously, our group developed epitope‐imprinted polymers (eIPs) for detection of AdV in tap water and human serum samples.^[^ ^23^ ^]^ The eIPs were synthesized using a functional monomer selected among 5 candidates by computationally investigating H‐bond interactions with the AdV template. The eIP‐based sensing platform achieved high affinity (K_D_ = 6.48 pm) and a detection limit of 10^2^ pfu mL^−1^ (0.2 fm) in real samples. The CIRs developed in this work were comprehensively designed, considering Coulomb and Lennard‐Jones potentials, alongside H‐bonding and minimum interatomic distance analysis. This enhanced accuracy in predicting T‐FM interactions, ultimately boosting CIR performance. Such a detailed design approach yielded high‐quality synthetic receptors without large aggregates, enhanced sensitivity within LOD of 0.064 fm, and improved affinity with K_D_ of 1.66 pm compared to eIPs. Furthermore, compared to eIPs, CIRs demonstrated high structural stability even after regenerative treatment, allowing for further sophisticated applications such as bioselective virus removal.

#### 2.3.2 Real Sample Analysis

Detection of waterborne pathogenic viruses is crucial for water quality management and disease prevention. The CIR‐conjugated piezoelectric sensor developed herein aims to provide a PoC monitoring tool for HAV detection in real samples. In order to demonstrate this application, CIR‐1@QCM surface was utilized for HAV determination in tap water and human serum, since CIR‐1 depicted better affinity and selectivity than CIR‐2. CIR‐1 could achieve the same LOD (i.e., 0.064 fm) in 50% tap water as buffer and demonstrated a steadily increasing ∆F as the HAV concentration increased from 0.064 fm to 64 pm (Figure 6H). In general, responses obtained for the tap water assay were higher than those obtained in PBST buffer, which can be attributed to nonspecific binding of interfering moieties present in the tap water. A linear correlation was observed between ∆F and virus concentration (∆F (Hz) = 2.113 Log *C*_HAV_ (pm) + 10.520) with R^2^ of 0.98 for the concentration range of 0.064 fm – 64 pm.

Similarly, HAV recognition performance of CIR‐1 was investigated in human serum (Figure 6I). It was observed that human serum, without any addition of HAV particles, caused a significant frequency decrease of approximately 30 Hz due to nonspecific binding of serum proteins onto the sensing surface. This led to an overall limited sensor response across the working range compared to PBST sensor output. Despite the suppression caused by the serum itself, CIR‐1 could still recognize the lowest virus concentration demonstrating a great sensitivity even in complex medium. Moreover, a calibration curve (i.e., Frequency change (Hz) = 0.508 Log *C*_HAV_ (pm) + 7.729) was obtained with R^2^ of 0.93.

The results of real‐sample investigations were subjected to two‐way ANOVA, along with HAV detection results obtained in buffer. The obtained F‐ value of 1.90 and the p‐value of 0.19 clearly indicated that the sensor performance is not significantly affected by the presence of impurities within the sensing environment. The statistical findings further highlight that CIR‐1 could reliably recognize HAV particles regardless of the media. This high sensitivity of the artificial receptor in a challenging detection environment (e.g., serum and tap water) was enabled by the data‐driven construction of the ligand, facilitating the selection of monomers that exhibit the strongest and most stable interactions with the epitope. As a result, a great sensitivity was empirically achieved even in real samples.

### 2.4 Membrane Application

Membrane technology has gained popularity worldwide for wastewater treatment due to its high separation efficiency, affordability, environmental friendliness, ease of use and maintenance, and relatively small footprint.^[^ ^29^ ^]^ In this study, the HAV‐specific CIRs were integrated with the PVDF and PES ultrafiltration membranes to achieve HAV removal with high selectivity and sensitivity. Prior to the membrane performance experiments, investigations on membrane surface characteristics were conducted to gain insights into their hydrophilicity upon each modification step, since elevated hydrophilicity improves virus filtration efficiency by increasing permeate flux and reducing membrane fouling. Additionally, extensive microscopic measurements were performed to investigate the structural features of the prepared membranes.

#### 2.4.1 Contact Angle and Swelling Degree Measurements

The hydrophilicity of the membranes after each functionalization step was investigated by calculating their swelling degree and evaluating goniometer images. The degree of swelling (DS) for each membrane type was quantitatively measured using the following formula (Equation 1):^[^ ^30^ ^]^

(1) $$Q=\frac{1}{\rho s}\frac{Wwet-Wdry}{Wwet}$$

where *Q* is the DS, *ρ_s_* is the solvent's density, and *W_dry_* and *W_wet_ *are the weights before and after adding the solvent to the membrane surface.

**Table** **1** represents the degree of swelling of bare, chitosan‐functionalized and CIR‐1 and CIR‐2 immobilized PVDF and PES membranes. DS was calculated based on Equation 1 by using the weights of membranes during each functionalization step, before and after adding 1.0 mL of water. Bare PVDF membrane showed the lowest DS since bare PVDF membrane has a hydrophobic nature. DS increased significantly after each modification step indicating improved hydrophilicity of PVDF membrane after each functionalization step. However, CIR‐1/Chi@PVDF membrane showed slightly larger hydrophilicity compared to the CIR‐2/Chi@PVDF. In contrast to PVDF membrane, bare PES membrane has a hydrophilic nature leading to instant absorption of the water droplet placed onto the surface. Therefore, DS calculations were more informative than goniometer investigations to understand their surface features after each modification. As a result, after chitosan functionalization and CIR immobilization, the hydrophilicity of the membrane was slightly enhanced. Similarly, the hydrophilicity of PES membrane improved significantly after CIR‐1 immobilization compared to CIR‐2.

**Table 1:** Degree of swelling (DS) and associated weights of PVDF and PES membranes in every functionalization step.

Membrane type ^a)^	W _dry_ [mg]	W _wet_ [mg]	DS ^b)^
Bare PVDF	65.60± 0.98	93.90 ± 1.95	0.30 ± 0.01
Chi@PVDF	69.70 ± 1.71	122.00_± 1.56	0.43_± 0.01
CIR‐1/Chi@PVDF	92.50 ± 1.62	178.70 ± 1.43	0.48 ± 0.01
CIR‐2/Chi@PVDF	86.23 ± 0.73	154.00 ± 1.15	0.44 ± 0.01
Bare PES	43.10 ± 0.79	79.32_± 1.16	0.46 ± 0.01
Chi@PES	45.66 ± 1.23	104.20 ± 0.95	0.56 ± 0.02
CIR‐1/Chi@PES	66.90 ± 1.05	161.50 ± 1.98	0.59 ± 0.01
CIR‐2/Chi@PES	74.70 ± 0.93	164.20 ± 1.32	0.55 ± 0.02

^a)^ Chi@PVDF: Chitosan‐functionalized PVDF, CIR‐1/Chi@PVDF: Chitosan‐functionalized PVDF after CIR‐1 immobilization, CIR‐2/Chi@PVDF: Chitosan‐functionalized PVDF after CIR‐2 immobilization, Chi@PES: Chitosan‐functionalized PES, CIR‐1/Chi@PES: Chitosan‐functionalized PES after CIR‐1 immobilization, CIR‐2/Chi@PVDF: Chitosan‐functionalized PES after CIR‐2 immobilization;

^b)^ DS: Degree of swelling; W_dry_ and W_wet_: the weight before and after adding the solvent to the membrane surface, respectively (Equation 1).

From a statistical perspective, Bartlett's test for DS revealed a test statistic of 14.7255 and a *p*‐value of 0.0397, indicating a statistically significant difference in variance of DS rates across all membrane types. Additionally, the analysis of coefficient of variation (CV) across membrane types provided insights into the variability of the membranes’ physical properties relative to their core trends (means) upon specific functionalizations. For dry weight, CIR‐2/Chi@PVDF and CIR‐2/Chi@PES showed the lowest CVs of 0.0085 and 0.0125, respectively, suggesting consistency and minimal variability, as compared to the other membranes. Similarly, the wet weight measurements for CIR‐2/Chi@PVDF (0.0075) and CIR‐1/Chi@PVDF (0.0080) demonstrated elevated stability, while bare PVDF and PES membranes indicated greatest variability (of 0.0208 and 0.0146), further highlighting the positive impact of CIRs immobilization on water retention and membranes’ structural integrity. In terms of DS, membranes with CIR modifications generally provided lower CVs across all measured conditions, reflecting enhanced membranes’ stability and consistency in their properties. Lower CVs in wet and dry weights, especially for CIR‐1/Chi@PVDF and CIR‐2/Chi@PVDF, indicate more uniform structural and water retention behavior. CIR‐1/Chi@PES has the lowest DS CV (see Table S2, Supporting Information), reflecting a stable swelling response, crucial for maintaining consistent pore sizes and ensuring effective virus retention. Compared to bare and chitosan‐functionalized membranes, modified membranes exhibit better control over swelling and structural stability, which enhances their suitability for virus filtration. These findings highlight CIR‐1′s role in stabilizing membrane properties and improving virus retention performance.

**Figure 7:**
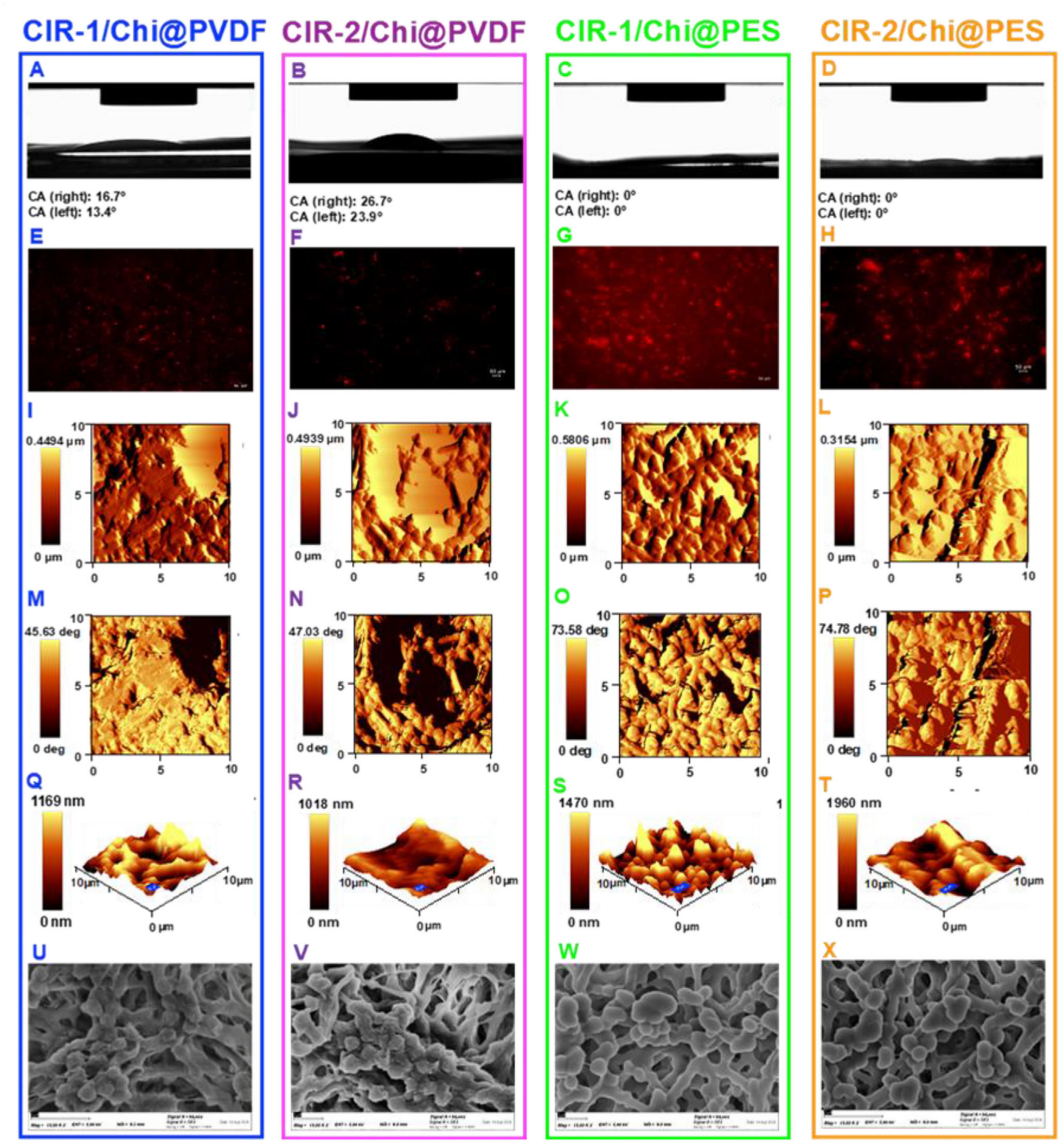
Goniometer images and contact angles A–D), fluorescence microscopy images E–H), AFM micrographs 2D height images I–L), phase images M–P), 3D topology Q–T) in 10 × 10 of µm scale, and SEM micrographs at 15000 × magnification (U‐X) of CIR‐1/Chi@PVDF, CIR‐2/Chi@PVDF, CIR‐1/Chi@PES and CIR‐2/Chi@PES.

Contact angle measurements were conducted using an optical contact angle meter on PVDF and PES membranes after each functionalization step. **Figure** **7**A–D shows the goniometer images and the membrane water contact angles (WCAs) upon different steps of membrane functionalization. Since bare PVDF has a hydrophobic nature, the water droplet was not absorbed by the membrane surface yielding the highest contact angle. However, the PVDF hydrophobicity decreased significantly after chitosan functionalization (Figure S18, Supporting Information). Moreover, the immobilization of CIRs further improved the water compatibility of chitosan functionalized PVDF membranes, particularly upon CIR‐1 immobilization, causing water droplets to be readily absorbed by the membrane. Figure 7B,D illustrates that the polymeric matrix of the CIR‐2 exhibits a more hydrophobic nature compared to CIR‐1. This observation is consistent with the swelling behavior study, where CIR‐2/Chi@PVDF showed a lower degree of swelling than CIR‐1/Chi@PVDF. In contrast, PES membranes maintained their hydrophilic nature throughout all functionalization steps. Water droplets were absorbed immediately by all PES membranes, resulting in WCAs measured as zero.

These findings highlight the tunable hydrophilicity and water compatibility of functionalized PVDF and PES membranes, demonstrating their potential for tailored applications in water filtration and virus retention upon HAV‐specific CIRs functionalization.

#### 2.4.2 Fluorescence Microscopy Analysis

The fluorescence image analysis of PVDF and PES membranes after each functionalization step was performed using a 10X objective lens of fluorescence microscopy (FLM; BZ‐X, Keyence, Neu‐Isenburg, Germany) to visualize CIR functionalization onto the membrane surfaces. In order to achieve this, both CIR‐1 and CIR‐2 polymerization mixtures included a rhodamine dye. Figure S19 (Supporting Information) illustrates the fluorescence microscopy images of bare and chitosan‐functionalized PVDF and PES membranes, respectively. The bare and chitosan‐functionalized membranes did not show any fluorescence since their chemical structures do not have any fluorescent properties. Figure 7E–H shows the CIR‐1/Chi and CIR‐2/Chi functionalized membranes, exhibiting significant fluorescence due to the rhodamine dye‐containing monomer. The CIR‐1/Chi@PVDF showed significantly stronger fluorescence emission than the CIR‐2 counterpart. A similar trend was observed in CIR‐1/Chi@PES and CIR‐2/Chi@PES. Furthermore, both CIR‐1/Chi@PES and CIR‐2/Chi@PES membranes revealed brighter fluorescence and a more pronounced red background than the PVDF ones, since PES has more hydrophilicity and swelling capacity allowing deeper incorporation of the CIR solution into the membrane's porous structure.

#### 2.4.3 Atomic Force Microscopy Analysis

The surface topology of bioselective PVDF and PES membranes after CIR immobilization was characterized using AFM at 10 µm × 10 µm scanning area. The 2D height images, phase images, and three‐dimensional (3D) surface topology images of membrane samples are illustrated in Figure 7I–T. The RMS roughness values of each 2D topology micrograph were calculated (**Table** **2**). Since the increase in the RMS roughness values indicates a more homogeneous distribution of ligands on membrane surface, CIR‐1 polymerization mixture was more effectively immobilized onto both Chi@PVDF and Chi@PES membranes, as indicated by their higher RMS values.

**Table 2:** RMS roughness values for 10 µm × 10 µm area of membrane samples.

Membrane type ^a)^	RMS [nm]
CIR‐1/Chi@PVDF	120.830
CIR‐2/Chi@PVDF	115.545
CIR‐1/Chi@PES	176.349
CIR‐2/Chi@PES	78.5275

^a)^ CIR‐1/Chi@PVDF: Chitosan‐functionalized PVDF after CIR‐1 immobilization, CIR‐2/Chi@PVDF: Chitosan‐functionalized PVDF after CIR‐2 immobilization, CIR‐1/Chi@PES: Chitosan‐functionalized PES after CIR‐1 immobilization, CIR‐2/Chi@PES: Chitosan‐functionalized PES after CIR‐2 immobilization.

The 2D height images revealed that CIR‐1/Chi@PVDF slightly more homogenous surface with a visible porous structure than CIR‐2/Chi@PVDF. Their phase images exhibited a similar phase degree, indicating their similar distribution of chemical phases on the surface. Furthermore, CIR‐1/Chi@PES showed the maximum height in the height images, where the scale bars beside the micrographs. Based on both 2D height and 3D topology images, CIR‐1/Chi@PES exhibited the most uniform distribution of chemical phases on the surface.

#### 2.4.4 Scanning Electron Microscopy Analysis

The morphological characteristics of CIR‐1/Chi@PVDF, CIR‐2/Chi@PVDF, CIR‐1/Chi@PES and CIR‐2/Chi@PES membranes were analyzed by SEM (Figure 7U–X). Prior to SEM analysis, the non‐conductive membrane surfaces were coated with gold layer via sputtering. CIR‐1/Chi@PVDF membrane resulted in sharper edges and larger cracks within the gold layer, where blob‐like features became more isolated compared to those seen with CIR‐2/Chi@PVDF. This trend was also observed in the comparative analysis of CIR‐1 and CIR‐2 immobilized PES membranes. The changes in microstructures may be attributed to the hydrophilicity of CIR polymeric composition on the membrane's surface that may have caused stress or restructuring, resulting in sharper edges and more pronounced cracks in the gold layer.^[^ ^31^ ^]^ Furthermore, SEM micrographs of CIR‐1/Chi@PES membrane showed a more pronounced background than the CIR‐2/Chi@PES, similarly with their corresponding fluorescence microscopy images, since PES is more hydrophilic and has a greater swelling capacity.

As a result of the thorough physico‐chemical characterization of bioselective membranes, a significant increase in surface hydrophilicity was observed by chitosan functionalization due to its hydrophilic nature. Further enhancement in hydrophilicity was achieved upon CIR immobilization. WCAs of functionalized membranes proved that polymerization mixture CIR‐1 has more hydrophilic property than the CIR‐2 counterpart which was also confirmed by FTIR characterization of the polymeric receptors. The same trend was observed in the swelling behavior study for both PVDF and PES membranes after CIR immobilization.

#### 2.4.5 Uptake Capacity and Regeneration Studies

HAV removal performance of CIR conjugated membranes was investigated in terms of uptake capacity and regeneration efficiency. For this, an HAV calibration curve with a linear dynamic range from 0.028 to 64 pm which we established in our previous work was utilized.^[^ ^32^ ^]^ In this study, uptake capacity and regeneration efficiency experiments were carried out by filtering 10 mL solutions containing 4.5 pm of inactivated HAV under vacuum by using CIR‐1 and CIR‐2 functionalized PVDF and PES membranes. For each test, the solution was filtered through a fresh membrane sample (3 cm × 3 cm) positioned in a 10 mL Büchner funnel, which was connected to a vacuum pump to facilitate the collection of the permeate. Pre‐ and post‐filtration solutions were evaluated in order to calculate HAV concentration of permeates based on the calibration curve equation by using absorbance values obtained from UV‐Vis spectroscopy measurements.

**Figure 8:**
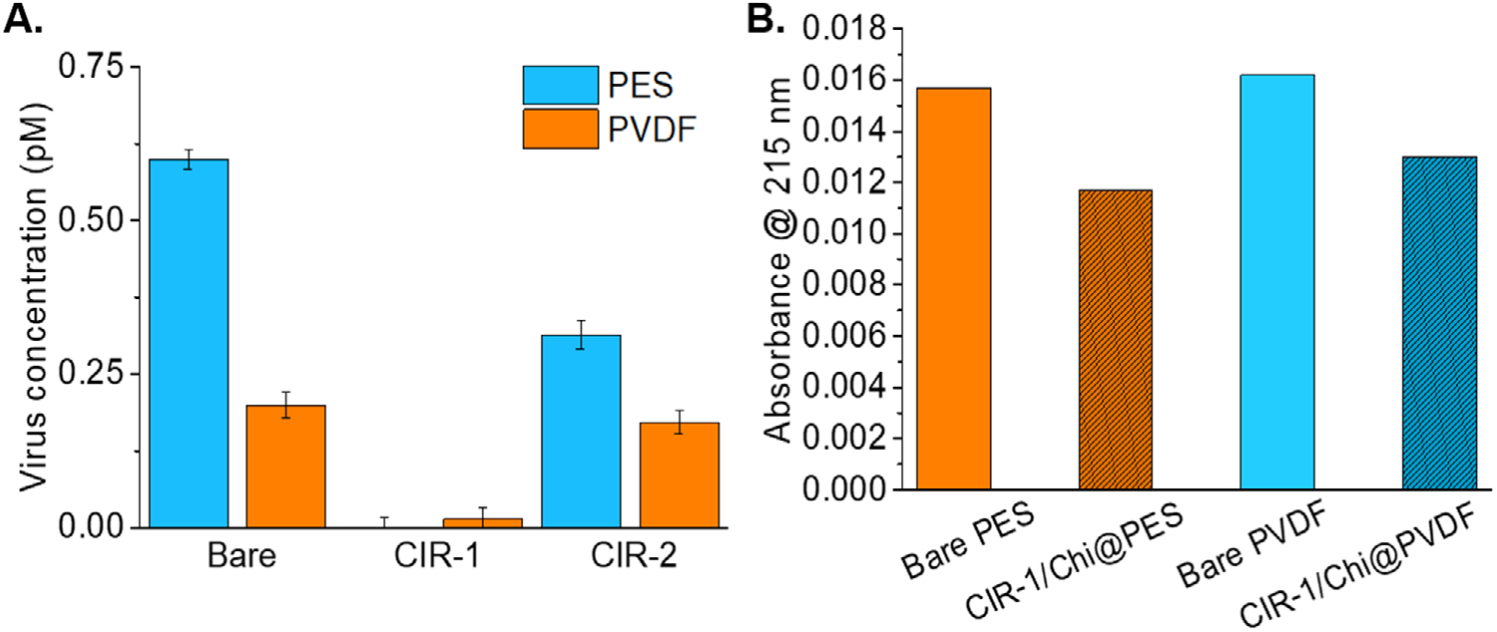
A) Comparison of HAV concentrations in initial loading solutions and permeates from bare and CIR‐1/Chi@PVDF, CIR‐1/Chi@PES, CIR‐2/Chi@PVDF, and CIR‐2/Chi@PES membranes. B) Comparison of absorbance values at 215 nm of bare PES, bare PVDF, CIR‐1/Chi@PVDF, and CIR‐1/Chi@PES membranes.

The results of membrane performance studies showed that both bare and functionalized membranes effectively reduced the filtrate's HAV concentration (**Figure** **8**A). Although bare ultra‐filtration membranes have inherent virus‐removal capabilities, significant amount of HAV remained in water even after filtration with bare PVDF (0.199 pm) and PES membranes (0.599 pm). The virus elimination capacity percentages, obtained from CIR‐2 immobilized membranes, especially for PVDF membranes, were similar to bare membranes. The poor virus elimination capacity of CIR‐2/Chi@PVDF may be attributed to the degrees of hydrophobicity of both PVDF membrane and CIR‐2′s polymerization mixture, as confirmed by WCA measurements. In contrast, CIR‐1/Chi@PVDF membrane effectively reduced HAV concentration yielding HAV removal up to 99.9% from contaminated water. Also, CIR‐1/Chi@PES membrane showed an excellent filtration performance, with 0.0% of the virus remaining in the filtrate at a loading volume of 10 mL.

The regeneration capacity of membranes was calculated at room temperature using 0.1 m HCl as a regeneration solution. Each membrane underwent a cycle of filtering 10 mL of the regeneration solution, followed by 10 mL of PBS. This cycle was repeated, and the PBS permeates from each cycle were analyzed using a UV‐vis spectrophotometer at the same wavelength range to assess the regeneration process effectiveness. HAV virus recovery (%) was calculated using Equation (2):

(2) $$\mathrm{HAV}\;\mathrm{virus}\;\mathrm{recovery}\left(\%\right)=\left(\frac{Cf}{Ci}\right)\times 100\%$$

where *C_f_* is the permeate concentration obtained after filtration [pm] and *C_i_* is concentration of loading HAV solution [pm].

**Table** **3** shows the virus recovery percentage after the regeneration process of functionalized PVDF and PES membranes. The filtrates obtained from filtration were analyzed using a UV‐vis spectrophotometer at 215 nm to measure the virus recovery rates after regeneration. The maximum virus recovery rate was 34.9% and 38.0%. Among all the tested membranes, CIR‐1/Chi@PVDF showed the highest virus recovery capacity. Similarly, an improved regeneration efficiency was also recorded in sensor applications for CIR‐1 conjugated QCM sensing platform, compared to CIR‐2 resulting in an improved virus binding performance for the HAV detection assay after regeneration (Figure S9E, Supporting Information).

**Table 3:** The virus recovery capacity after the regeneration of PVDF and PES membranes with filtration using 0.1 m HCl.

Membrane type ^a)^	Filtration order	Virus recovery [%]
CIR‐1/Chi@PVDF	1st 2nd	34.9 38.0
CIR‐2/Chi@PVDF	1st 2nd	1.2 4.8
CIR‐1/Chi@PES	1st 2nd	16.2 20.3
CIR‐2/Chi@PES	1st 2nd	1.7 –

^a)^ CIR‐1/Chi@PVDF: Chitosan‐functionalized PVDF after CIR‐1 immobilization, CIR‐2/Chi@PVDF: Chitosan‐functionalized PVDF after CIR‐2 immobilization, CIR‐1/Chi@PES: Chitosan‐functionalized PES after CIR‐1 immobilization, CIR‐2/Chi@PES: Chitosan‐functionalized PES after CIR‐2 immobilization.

#### 2.4.6 Human Serum Studies

Based on the comprehensive characterization and membrane performance evaluations, including uptake capacity and regeneration experiments, CIR‐1 was selected over CIR‐2, prompting the subsequent serum studies utilizing CIR‐1. Uptake capacity studies were conducted also in human serum (50% diluted with PBS) to assess the applicability of CIR‐1 conjugated membranes in HAV removal from complex medium. All of the human serum experiments were carried out for bare PES and PVDF, CIR‐1/Chi@PVDF, and CIR‐1/Chi@PES. First, 10 mL of 4.5 pM HAV concentration was prepared in 50% diluted human serum and filtered through bare PES and PVDF, CIR‐1/Chi@PVDF, and CIR‐1/Chi@PES membrane samples. The absorbance values of all membranes were measured by UV‐vis spectrophotometer at 215 nm. Figure 8B shows the results of membrane performance studies conducted in serum using both bare and CIR‐1 functionalized membranes. Both CIR‐1/Chi@PVDF and CIR‐1/Chi@PES reduced the filtrate's HAV concentration with greater efficiency compared to their bare counterparts. CIR‐1/Chi@PES showed slightly less absorbance value at 215 nm indicating better virus removal capacity, similar to uptake capacity study carried out in contaminated water. These findings demonstrated that CIR‐1 carrying membranes can be applied in sample pretreatment for chromatographic bioanalysis in which capturing and/or removal of HAV is required.

Lu et al. developed hierarchical‐microporous PVDF membranes with modifications like zinc oxide grafting for increased hydrophilicity, silver for antibacterial properties, and 3‐(trimethoxysilyl)propyl methacrylate for enhanced functionality. These membranes served as a platform for creating bisphenol A molecularly imprinted membranes (BPA‐MIMs), which efficiently removed BPA from water at concentrations below 75 mg L^−1^ within 60 min.^[^ ^10^ ^]^ In prior work, the same group developed pyrimethamine‐MIMs functionalized with carbon nanotubes for selective separation of pyrimethamine from wastewater. Photo‐initiated “click chemistry” polymerization created specific recognition sites on the membrane. These membranes were highly efficient in removing contaminants from wastewater.^[^ ^33^ ^]^ Schwark et al. developed epitope‐imprinted polymeric membranes targeting the C‐terminal fragment of immunoglobulin G (IgG) heavy chain for improved purification of monoclonal antibodies. They demonstrated high IgG affinity, achieving near‐pure IgG elution (95–100%), with a dynamic binding capacity of 3.9 mg mL^−1^. Additionally, the membranes effectively reduced host cell protein contamination by 88%.^[^ ^34^ ^]^

Earlier, our group developed bioselective membranes by combining chitosan functionalization and virus‐templated molecularly imprinted nanoparticles (nanoMIPs) to separate adenoviruses from water samples.^[^ ^35^ ^]^ However, the structural barriers of nanoMIPs on the surface can restrict the selective binding with the intended target. To overcome cross‐reactivity and whole‐molecule imprinting challenges, our group also demonstrated that conventionally synthesized eIPs can be immobilized on bioselective membranes, rendering them ideally compatible with covalent surface bonding.^[^ ^32^ ^]^ Previously developed bioselective membranes could eliminate viruses from water samples; however, those membranes face challenges in filtering trace levels of viruses. Moreover, conventionally synthesized eIPs did not allow efficient removing of HAV virus from highly complex media like serum. Herein, this study successfully addressed this challenge by combining the computationally designed virus‐specific imprinted receptor immobilization onto membranes, resulted in an efficient HAV removal from contaminated water with a 100% purification rate. Moreover, one quarter of the original virus concentration present in the loading solution was able to recover after regeneration for CIR‐1/Chi@PES. This demonstrated the effectiveness of integrating specific molecular recognition strategies with surface modification techniques in improving membrane filtration performance. CIRs offer superior selectivity by incorporating molecular recognition sites designed to target specific regions of virus structures. This targeted approach enhances viral clearance efficiency, minimizes secondary contamination risks, and addresses the challenges of whole‐virus imprinting. CIRs are particularly suitable for high‐purity applications, such as healthcare and advanced water treatment, where rigorous purification standards are essential.

## 3 Conclusion

This proof‐of‐concept study demonstrates the successful development of computationally designed CIRs to be applied in biosensing and removal of HAV particles in complex media. Comprehensive computational analyses were employed to identify epitope 2 (amino acid sequence: TTHALFH) as the most structurally stable, and dynamically exposed target during 1 µs MD productions. Molecular docking and MD studies further revealed that the best‐performing formulations for experimental validation were AMPSA‐BAP‐HEMA (CIR‐1) and MEP‐BAP‐NOBE (CIR‐2), both in 1:7 ratio. These formulations demonstrated favorable interaction energies of −114.13 kJ mol^−1^ for CIR‐1 and −90.21 kJ mol^−1^ for CIR‐2, highlighting their strong potential for experimental implementations. The empirical performance of CIRs was demonstrated in biosensing and membrane filtration applications which require virus‐specific ligands. Both CIR‐1 and CIR‐2 exhibited high sensitivity for HAV particles on the QCM platform, achieving a LOD of 0.064 fm. In comparative studies, CIR‐1 showed superior affinity and selectivity compared to CIR‐2. CIR‐1 exhibited a 12‐fold stronger affinity (K_D_ = 1.66 pm) than CIR‐2 (K_D_ = 20.40 pm). The increased affinity of CIR‐1 over CIR‐2 was linked to its improved kinetic behavior observed in MD trajectories, offering experimental validation of the computational analysis. Furthermore, CIR‐1 could maintain its high sensitivity even in complex detection media such as tap water and human serum. In addition, PVDF and PES membranes were successfully functionalized with CIRs and comprehensively characterized. Swelling behavior and water contact angle measurements revealed a significant enhancement in the hydrophilicity of membranes, particularly after CIR‐1 immobilization. The prepared membranes were analyzed using AFM and SEM to assess their surface morphology, phase distribution, and topography. Immobilization of computationally designed CIR‐1 on PVDF and PES membranes resulted in an efficient HAV removal from contaminated water with 99.99% and 100% purification rate for PVDF and PES, respectively. This demonstrated the effectiveness of integrating specific molecular recognition strategies with surface modification techniques in improving membrane filtration performance, even from highly complex media like serum. This work reveals that our holistic computational design approach yields to epitope‐specific artificial virus ligands with great sensitivity, affinity, and selectivity, allowing for the HAV detection and removal at ultra‐trace amounts even in biofluids. This integrated approach underscores the promise of computationally designed imprinted synthetic ligands in enhancing point‐of‐care diagnostics and water treatment technologies for virus detection and removal. Moreover, this rapid, eco‐friendly, and cost‐effective strategy holds significant potential for developing new CIRs to target and eliminate other pathogenic viruses.

## 4 Experimental Section

### Computational Section

Molecular docking studies were conducted for all 18 FMs interacting with two distinct epitopes, considered separately. The HADDOCK server^[^ ^36^ ^]^ was employed to dock the FMs against both epitopes, using vacuum conditions with both rigid and semi‐flexible iterations. The best binding poses, determined by the highest (most negative) binding energies and docking scores, were selected based on the largest cluster sizes and the most negative Z‐scores. For analysis purposes, PyMol^[^ ^37^ ^]^ and Chimera^[^ ^38^ ^]^ packages were used.

The 36 selected docked complexes were then subjected to 100 ns of classical MD simulations using the Gromacs package,^[^ ^39^ ^]^ in 3 replicas per system, with periodic boundary conditions (PBC) applied and without constraints. The epitope topologies were derived from the cryo‐EM structure of Hepatitis A (PDB: 5WTE^[^ ^18^ ^]^), while FMs’ coordinates were retrieved from PubChem database,^[^ ^40^ ^]^ and modeled using Chimera^[^ ^38^ ^]^ and Avogadro^[^ ^41^ ^]^ software. The monomers’ bonded and non‐bonded parameters were generated via the external force field module CGenFF,^[^ ^42^ ^]^ highly compatible with Charmm force field.^[^ ^43^ ^]^

The docked T‐FMs complexes were parameterized and simulated using CHARMM36 force field^[^ ^43^ ^]^ and solvated with the TIP3P water model.^[^ ^44^ ^]^ Systems were neutralized with counter‐ions and energy minimized using the Steepest Descent algorithm^[^ ^45^ ^]^ for 50 000 steps. Following minimization, systems underwent NVT (constant number of particles, volume, and temperature) equilibration at 300 K temperature and NPT (constant number of particles, pressure, and temperature) equilibration at 1 bar pressure for 1 ns, using the V‐rescale thermostat,^[^ ^46^ ^]^ Parrinello‐Rahman barostat,^[^ ^47^ ^]^ and LINCS algorithm^[^ ^48^ ^]^ for holonomic constraints. Long‐range electrostatics were handled using the Particle Mesh Ewald (PME)^[^ ^49^ ^]^ summation method with a 1.2 nm cutoff.

A total of 54 distinct systems in 3 replicas/system (162 MD trajectories) were analyzed using *gmx* tools, alongside PyMol^[^ ^37^ ^]^ and VMD^[^ ^20^ ^]^ for trajectory visualization, with additional statistical analyses and plots scripted in Python 3.8. The quantities of interest in our analyses include: binding affinities, surface energies, H‐bonds, minimum distances, Lennard‐Jones and Coulomb potentials, with an additional focus on total interaction energies. In terms of statistical analysis, we considered descriptive metrics such as mean, median, standard deviations, minimum and maximum values across specific distributions, two‐way ANOVA, and extensive correlations to determine relevant relationships between groups of data and/or particular features. Additionally, statistical analyses applied to the experimental data included, besides two‐way ANOVA,^[^ ^50^ ^]^ Kruskal‐Wallis tests,^[^ ^51^ ^]^ Spearman correlations,^[^ ^52^ ^]^ Cohen's d values,^[^ ^53^ ^]^ and coefficient of variation (CV) analysis.

### Experimental Section: CIR Synthesis and Characterization

The in‐silico designed polymeric ligands were synthesized via solid‐phase synthesis method.^[^ ^17^ ^]^ For this, 120 g glass beads (GBs) were utilized as solid support phase which were initially boiled in 1 m NaOH for 15 min and washed thoroughly. The hydroxyl functionalized beads were then silanized via overnight incubation with 2% v/v (3‐aminopropyl)trimethoxysilane (APTMS) in anhydrous toluene. Following the washing of the beads with acetone and methanol, the surface of GBs with further functionalized with 7% v/v glutaraldehyde solution in 10 mM PBS for 2 hours. For covalent immobilization of the template, 20 mg epitope (24.14 µmol) was solubilized in 50 mL PBS and incubated with functionalized GBs overnight at room temperature. The Schiff base bonds were reduced with 50 mg of NaBH_4_ in 50 mL of PBS for 30 min, and GBs were washed with deionized water. The unoccupied aldehyde groups on the beads were blocked by 50 mL 0.1 mm ethanolamine solution in PBS with 15 min incubation.

120 g epitope conjugated GBs were purged with N_2_ prior to polymerization reaction. Meanwhile, polymerization was prepared with functional monomers and ultrapure water (UPW) as solvent. The polymerization mixture for CIR‐1 included 22 mg (169.5 µmol) 2‐hydroxyethylmethacrylate (HEMA), 35.13 mg (169.5 µmol) 2‐acrylamido‐2‐methylpropane sulfonic acid (AMPSA), 36.82 mg (169.5 µmol) 2,6‐bis(acrylamido)pyridine (BAP) in 49 mL UPW. Meanwhile, CIR‐2 mixture was composed of 33.12 mg NOBE, 38.32 mg MEP, and 36.82 mg BAP in 49 mL UPW. Additionally, both recipes included 4 mg of methacryloxyethyl thiocarbamoyl rhodamine B (M‐Rho) to introduce fluorescent properties, and 55 mg N‐(3‐aminopropyl) methacrylamide hydrochloride (APMA) to obtain primary amine groups in the polymeric matrix to facilitate covalent immobilization onto the sensor and membrane surfaces. Once the polymerization mixture was prepared, the solution was sonicated for 30 min followed by 30 min of N_2_ purging to remove oxygen. The purged solution was mixed with epitope conjugated GBs and purged 20 min more and the polymerization reaction was initiated by addition of 48 mg of ammonium persulfate (APS) and 48 µL N,N,N′,N′‐tetramethylethylenediamine (TEMED) dissolved in 500 µL of UPW separately. The polymerization reaction took place at room temperature for 2 hours. After the completion of reaction, the GBs were transferred into 60 mL solid phase extraction columns with the polymerization mixture. Prior to collection of high affinity CIR nanoparticles at 65 °C, the low affinity particles and oligomers were removed with cold water washing step at 4 °C. The collected CIRs were freeze‐dried and aliquoted at desired concentration to be kept at −20 °C until further usage. Non‐imprinted polymers (NIPs) were synthesized for each recipe using the exact same synthesis method without the addition of the epitope template.

### Experimental Section:CIR‐QCM Sensor Fabrication and Characterization

First, the QCM crystals were cleaned for 5 min in 14 mL of basic piranha solution containing ammonia (25%), hydrogen peroxide (35%) and UPW at a volume ratio of 1:1:5. The cleaned crystals were treated with 2 mm MUDA solution in ethanol by overnight incubation at room temperature to obtain self‐assembled monolayer of MUDA on gold substrate. The MUDA‐coated crystal was assembled in the QCM‐I measurement chamber, and PBS was injected at 15 µL min^−1^ until the frequency signal is stabilized. Once the stable PBS signal was obtained, the carboxylic acid groups on the surface were activated with a mixture of 0.2 m EDC and 0.05 m NHS for 4 min at 15 µL min^−1^. 0.5 mg mL^−1^ of CIRs in immobilization buffer (i.e., 10 mm Sodium acetate at pH 5 or 10 mM MES buffer at pH 6) were injected into the chamber for 30 min at 10 µL min^−1^. After 30 min of CIR immobilization the chamber was treated with PBS again to remove unbound CIR particles and to observe the signal change resulting from CIR immobilization. The non‐conjugated functional groups on the glass substrate were blocked by 0.1 m ethanolamine treatment for 5 min at 15 µL min^−1^ to prevent nonspecific binding.

### Experimental Section:Virus Binding Assays

Epitope detection studies of CIR‐1 were performed for the epitope concentration range of 0.1–1000 µm. Epitope solutions were prepared in PBS by 10‐fold serial dilution of 1 mm stock epitope solution. Similarly, the epitope binding performance of CIR‐2 was examined for samples with epitope concentration range of 1–1000 µm prepared by serial‐dilution of 1 mm stock solution. The assays were performed by initially injecting PBS running buffer onto prepared sensing substrate to obtain a reference point at 15 µL min^−1^, followed by sequential injection of epitope sample solutions starting from lowest to highest concentration value. For each concentration, the initial injection rate was set at 1.90 mL min^−1^ for approximately 20s to flush away the previously injected solution, followed by a reduction to 15 µL min^−1^ for 10 min until stabilization. The frequency difference (∆F) was calculated by subtracting the average sample frequency from the reference frequency value.

HAV binding assays were performed for both CIR‐1 and CIR‐2 were performed for a virus concentration range of 0.064 fm –64 pm which were prepared by 10‐fold serial dilution in running buffer. A 10 mm PBS containing 0.05% (v/v) Tween 20 (PBST) was used as the running buffer for binding assays of CIR‐1. Meanwhile, 10 mm PBS was used as running buffer for binding experiments of CIR‐2 since the surfactant inhibited virus binding at lower concentrations. For both assays, running buffer was injected to obtain a reference frequency value. After stable reference point was obtained virus solutions were sequentially injected starting with the lowest concentration. Between the virus injections, the weakly bound viruses were removed with a rapid PBST wash at 1.90 mL min^−1^. The sensor response ∆F was calculated by subtracting the average sample frequency value from the average reference frequency at each sample concentration.

Selectivity was investigated by replacing CIR‐1 and CIR‐2 with NIP‐1 and NIP‐2 during the sensor fabrication process. Cross‐reactivity of CIRs was investigated by performing virus binding assays via replacing HAV with non‐targeted viruses (i.e., HSV and AdV).

Affinity analysis was performed with Biacore X100 SPR system with gold SPR chips functionalized with a 2 mm 11‐Mercaptoundecanoic acid solution. 0.5 mg mL^−1^ CIRs in optimum immobilization buffer were incubated for 30 min, following a 4‐minutes surface activation with a 0.2 m EDC and 0.05 m NHS solution. To block any unoccupied sites, the sensor was treated with 0.1 m ethanolamine. HAV samples, prepared in 10 mm phosphate‐buffered saline containing 0.05% Tween, were injected for 9 min at concentrations ranging from 0.064 to 640 pm, with a 120‐second dissociation phase. The resulting sensogram was processed using Biacore X100 software to assess real‐time kinetics. The sensor performance of CIR‐1 was assessed using spiked tap water and human serum samples to simulate real‐world conditions. For tap water, HAV was spiked at concentrations of 0.064 fm – 64 pm in 50% tap water diluted with PBST. A reference solution (unspiked tap water‐PBST, 1:1 v/v) was injected and monitored until stabilization.

For human serum experiments, serial dilutions were made to obtain HAV spiked samples with a concentration range of 0.064 fm – 64 pm. Human serum solution without HAV served as the reference. Spiked samples were injected into the CIR‐1‐QCM sensor after a PBST wash and monitored for 10 min at room temperature starting with the lowest concentration.

### Experimental Section:Chitosan Functionalized and CIR Immobilized PVDF and PES Membranes

A 1.0 wt.% chitosan solution was freshly prepared in 2.0 wt.% acetic acid diluted in distilled water, vortexed, and sonicated for 30 min to enhance its homogeneity. Commercial PVDF and PES membranes (3 cm × 3 cm) were initially moistened with distilled water for 5 min and then submerged in the chitosan solution for 2 min to ensure adequate coating. In the next step, the membranes were heated at 60 °C for 45 min under vacuum‐drying conditions. The dried PVDF and PES membranes were then immersed in a 1.0 m NaOH solution within a 50% (v/v) water‐ethanol mixture for 30 min for neutralization. Following this, the samples were rinsed with a 50% (v/v) ethanol–distilled water mixture (10 min) and distilled water (30 min), respectively. The membranes were dried in a vacuum desiccator for 30 min.^[^ ^35^ ^]^ For the immobilization of computationally designed CIRs, chitosan functionalized PVDF and PES membrane samples were initially treated with a 5% (v/v) aqueous GA solution for 15 min. Subsequently, 200 µL of CIR dispersion was applied to the membrane surface, followed by a 30‐min incubation. The membranes were dried for an additional 30 min in a desiccator under vacuum conditions.

### Experimental Section:Virus Filtration and Regeneration Studies

Virus filtration experiments were conducted to assess uptake capacity and human serum interactions by filtering 10 mL solutions containing 4.5 pm of inactivated HAV. In each test, the solution was passed through a new CIR‐1 or CIR‐2 immobilized membrane sample (3 cm × 3 cm), placed in a 10 mL Büchner funnel connected to a vacuum pump to collect the permeate. The funnel was positioned above a filtration flask linked to the vacuum pump (at 0.03 mbar), which collected the filtrate ‐ the solution that passed through the membrane. Pre‐ and post‐filtration solutions were analyzed to determine HAV concentrations in the permeates, using the calibration curve equation derived from absorbance measurements obtained by UV‐vis spectroscopy.

For regeneration studies, the membrane's regeneration capacity was evaluated at room temperature with regeneration solutions of 0.1 m HCl, ACN (at 15 °C for the first filtration and 60 °C for the second), and 0.1 m NaOH. Each membrane underwent a cycle of filtering 10 mL of regeneration solution followed by 10 mL of PBS. This cycle was repeated, and the PBS permeates from each cycle were analyzed using UV‐vis spectroscopy at the same wavelength range to assess the effectiveness of the regeneration process. Permeate concentrations before and after regeneration were calculated based on the calibration curve equation using the absorbance values obtained from UV‐vis measurements.

### Experimental Section:Statistical Analysis

For dose‐dependent response analyses of CIR‐1 versus NIP‐1 and CIR‐2 versus NIP‐2, and direct comparison between CIR‐1 versus CIR‐2′s rebinding, concentration ranges were log‐transformed (log₁₀) to stabilize variances and linearize relationships for ANOVA. No outlier removal or additional normalization of sensor signal data was performed. Each group of interest consisted of 7 variables corresponding to 7 analyte concentrations. Statistical analyses included two‐way ANOVA for particle type versus peptide concentration range. As a complementary approach, Kruskal‐Wallis tests (nonparametric, two‐sided, α = 0.05) were considered to assess if the observed differences in sensor signal distributions between, e.g., CIR‐1 and NIP‐1, are indeed statistically significant. Spearman correlation was used to evaluate monotonic relationships between concentration and sensor signal, and Cohen's d to estimate the effect sizes, as p‐values alone do not state the magnitude of observed effects. No post‐hoc tests or α adjustments were applied. All data were visualized using boxplots for direct group comparisons. The statistics related to CIR's specificity required no data transformations or normalization steps. Data are represented as mean ± SD for each analyte type (HAV/AdV/HSV, n = 2 per analyte). The test for differences in mean sensor signals among the analytes, was carried out using two‐sided one‐way ANOVA (α = 0.05), with homogeneity of variances confirmed via Levene's test (α = 0.05). Additionally, pairwise post‐hoc comparisons were conducted using Tukey's HSD method (α = 0.05).

For the degrees of swelling assessment of the CIR‐functionalized PVDF and PES membranes, all variables were considered as mean ± SD, upon which coefficient of variation (CV = SD/mean) method was used to express relative variability among membranes and functionalization conditions. Each membrane formulation was measured in n = 3 independent samples for dry weight (W_dry), wet weight (W_wet), and degree of swelling (DS), in 8 different conditions. Raw replicate values were screened for outliers with no data points excluded, nor imputed. The homogeneity of variances across eight membrane formulations was also evaluated via Bartlett's test (df = 7, α = 0.05). All statistics were carried out in Python (version 3.8.10) using data handling with pandas, SciPy for ANOVA and variance analysis, statsmodels for Tukey's HSD, as well as matplotlib and seaborn for plotting.

## Conflict of Interest

The authors declare no conflict of interest.

## Author Contributions

E.A. and E.S. contributed equally to this work. N.S.M.C. carried out molecular docking and molecular dynamics simulations and performed statistical data evaluation; E.A. designed and conducted membrane characterization and virus filtration experiments and performed Raman, XPS, FTIR, AFM, contact angle, and fluorescence microscopy measurements; E.S. designed and conducted QCM sensor characterization and virus detection experiments, as well as DLS, ELS, and fluorescence microscopy measurements; S.T. carried out QCM sensor experiments; S.S. and R.S. contributed to epitope synthesis and characterization; F.J. and M.G. synthesized and characterized BAP and NOBE monomers; E.A., N.S.M.C., and E.S. wrote the initial manuscript draft; Z.A. conceived the idea, administered the project, acquired funding, contributed to conceptualization, designed the experiments, supervised the research, and edited and finalized the manuscript.

## Supporting information



Supporting Information

## Data Availability

The data that support the findings of this study are available from the corresponding author upon reasonable request.
