# Supplementary material for: Atomic‐Level Engineering of Synthetic Receptors for Enhanced Virus Detection and Removal
Source: Adv Healthc Mater. 2025 Aug 25;14(25):2502043. doi: 10.1002/adhm.202502043 (PMC12477568; doi:10.1002/adhm.202502043)
Supplement: Supplementary file 1 — Supporting Information [file ADHM-14-0-s001.pdf]

ADVANCED HEALTHCARE MATERIALS

Supporting Information

for *Adv. Healthcare Mater.*, DOI 10.1002/adhm.202502043

Atomic-Level Engineering of Synthetic Receptors for Enhanced Virus Detection and Removal

*Eda Akin, Ekin Sehit, Nastasia Sanda Moldovean-Cioroianu, Sahana Tavaragondi, Sophia Slenczka, Roderich Süssmuth, Friedrich Jurk, Manuel van Gemmeren and Zeynep Altintas**

ADVANCED HEALTHCARE MATERIALS

SUPPORTING INFORMATION

Atomic-Level Engineering of Synthetic Receptors for Enhanced Virus Detection and Removal

Eda Akin[1]†, Ekin Sehit[1]†, Nastasia Sanda Moldovean-Cioroianu[1], Sahana Tavaragondi[1], Sophia Slenczka[2], Roderich Süssmuth[2], Friedrich Jurk[3], Manuel van Gemmeren[3], Zeynep Altintas[1,4]*

[1]Institute of Materials Science, Kiel University, 24143 Kiel, Germany

[2] Institute of Chemistry, Technical University of Berlin, 10623 Berlin, Germany

[3] Otto Diels-Institute of Organic Chemistry, Kiel University, 24118 Kiel, Germany

[4] Kiel Nano, Surface and Interface Science (KiNSIS), Kiel University, 24118 Kiel, Germany

† Equally contributing first authors

*Corresponding author: Z. Altintas

E-mail: zeynep.altintas@tf.uni-kiel.de

Table of Contents

Figure S1. Correlation heatmap between energy components for epitope 1 vs epitope 2. **S-4**

Table S1. Binding affinities of FMs docked against both HAV epitope1 and epitope 2**.S-5**

Figure S2. Structure of HAV epitope 2 (THR70-HIS76) with 21 active sites: 2 O atoms from hydroxyl groups (green), 1 O atom from carboxylic group (blue),7 O atoms from six amide groups and one carboxyl group (red), 1 C atom from benzyl group (brown),6 N atoms from six amide groups (purple),1 N atom from primary amine group (pink),4 N atoms from heterocyclic amine groups (yellow and black); however, one of them is not available due to the steric hindrance. **S-6**

Reagents and chemicals S-6

Instrumentation S-7

Synthesis of N,O-bismethacryloyl ethanolamine (NOBE) S-8

Figure S3. Synthesis procedure of NOBE monomer. **S-8**

Figure S4. NMR analysis of in-house synthesized NOBE monomer. **S-9**

Synthesis of 2,6-bis(acrylamido) pyridine (BAP) S-10

Figure S5. Synthesis procedure of BAP monomer. **S-10**

Figure S6. Resulting NMR spectrum of in-house synthesized BAP monomer. **S-11**

Figure S7. FTIR spectra of NOBE (A) and BAP (B) monomers. **S-12**

Characterization of monomers S-12

Synthesis of Hepatitis A virus epitope S-12

Figure S8. HRMS (A and B) and NMR (C) characterization of in-house synthesized HAV epitope 2. **S-14**

Ninhydrin test S-15

Figure S9. Colors of ninhydrin test before (left) and after (right) epitope incubation. **S-15**

Figure S10. Hydrodynamic size and zeta potential of (A, B) CIR-1 and (C, D) CIR-2, respectively. **S-16**

Figure S11. Hydrodynamic size and zeta potential of (A, B) NIP-1 and (C, D) NIP-2 respectively. **S-17**

Figure S12. (A) Raman spectrum of CIR-2 together with its functional monomers. XPS white spectra of (B) CIR-1 and (C) CIR-2. **S-18**

Figure S13. XPS results for MUDA coated gold QCM substrate with (A) white. (B) carbon, (C) oxygen, and (D) nitrogen spectra. **S-19**

Characterization of CIRs S-19

Figure S14. Fluorescence micrographs for MUDA, CIR-1 and CIR-2 functionalized QCM substrates. **S-21**

Virus concentration conversion S-21

Equation S1. Sauerbrey equation showing the effect of adsorbed mass on the resonance frequency change of piezoelectric crystal. **S-22**

Figure S15. Concentration dependent frequency change of CIR-2 conjugated QCM crystal for HAV epitope range of 1 - 1000 µM (A). CIR-1 (B) and CIR-2 (C) HAV sensing response when two different immobilization buffers were utilized for ligand conjugation. Frequency change of CIR-2 conjugated QCM sensor for HAV concentration range of 0.064 fM - 64 pM with logarithmic regression analysis (D). HAV detection performance of CIR-1 (E) and CIR-2 (F) conjugated sensing platforms before and after regeneration with basic treatment. **S-23**

Figure S16. Fluorescence micrographs of CIR-1 and CIR-2 functionalized QCM substrates before and after basic treatment for regeneration. **S-24**

Figure S17. HAV detection with CIR-2 and control non-imprinted polymer (NIP-2) synthesized without epitope template. **S-24**

Figure S18. Goniometer images for contact angles (CAs) of bare PVDF and PES membranes, Chi@PVDF and Chi@PES. **S-25**

Table S2. Coefficient of variation (CV) analysis across CIR-1 vs CIR-2 functionalized PVDF and PES membranes. **S-25**

Figure S19. Fluorescence microscopy images of bare PVDF and PES membranes, Chi@PVDF and Chi@PES. **S-26**

References S-27

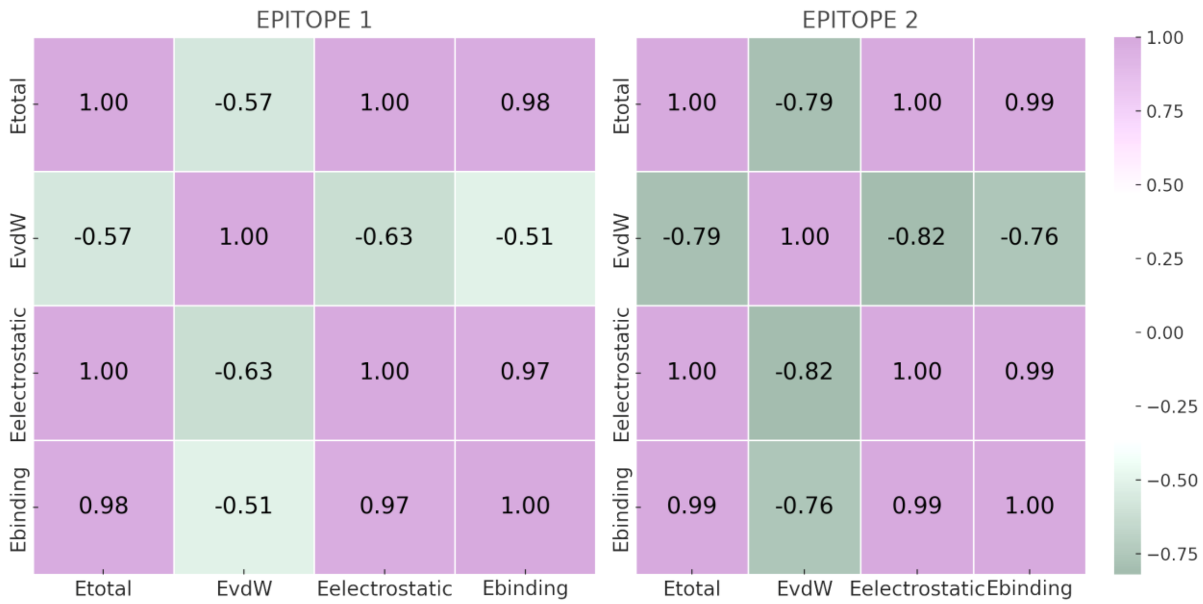

Figure S1. Correlation heatmap between energy components for epitope 1 vs epitope 2.

For both epitopes, electrostatic interactions are the dominant contributors to the total binding energy, with a near-perfect positive correlation. Binding energy also shows strong positive correlations with total and electrostatic energies (>0.97), highlighting their critical role in binding stability. Van der Waals interactions exhibit moderate-to-strong negative correlations with total and electrostatic energies, with a stronger influence observed in epitope 2.

Overall, the results underscore the primary importance of electrostatic interactions, with van der Waals forces providing additional but varying contributions across epitopes.

Table S1. Binding affinities of FMs docked against both HAV epitope1 and epitope 2.

FM type	FM of interest	Epitope 1 - seq: NYNHSDEY				Epitope 2 - seq: TTHALFH			
		E_{total} [kcal/mol]	E_{vdW} [kcal/mol]	$E_{electrostatic}$ [kcal/mol]	$E_{binding}$ [kcal/mol]	E_{total} [kcal/mol]	E_{vdW} [kcal/mol]	$E_{electrostatic}$ [kcal/mol]	$E_{binding}$ [kcal/mol]
Acidic	MAA	-83.393	-6.103	-78.430	-59.572	-108.521	1.372	-109.894	-101.418
	AcA	-54.027	0.348	-54.376	-48.589	-125.083	2.857	-127.940	-102.402
	ITA	-107.021	-4.716	-102.304	-85.320	-170.530	-0.611	-169.918	-144.056
	TfMAA	-53.291	-9.326	-44.687	-40.532	-84.872	-4.125	-81.312	-78.884
	PVA	-59.638	-12.460	-47.896	-44.246	-92.798	-1.777	-91.021	-87.549
	AMPSA	-52.038	-7.699	-44.453	-51.209	-88.138	-2.307	-86.931	-74.293
	MEP	-83.586	-7.301	-82.154	-80.176	-55.634	-5.535	-50.098	-55.238
Basic	4VP	-21.806	-12.349	-11.339	-23.479	-20.627	-8.280	-12.643	-24.733
	DAM	-128.149	-6.433	-125.206	-120.457	-79.888	-8.117	-72.141	-80.168
	DEM	-127.727	-6.113	-122.179	-121.478	-82.593	-8.119	-75.722	-78.453
	PAS	-17.267	-13.824	-5.105	-22.507	-17.821	-12.792	-5.046	-18.543
	1VI	-24.740	-10.906	-14.082	-25.778	-22.455	-7.779	-14.848	-24.980
	4VI	-27.167	-10.189	-17.264	-29.089	-32.489	-9.071	-23.564	-30.878
	BAP	-36.311	-16.395	-21.430	-42.034	-58.845	-13.286	-46.960	-57.998
Neutral	AA	-33.912	-8.076	-27.578	-34.501	-39.324	-7.671	-31.727	-37.828
	MA	-37.532	-9.247	-28.285	-33.709	-35.177	-7.672	-28.632	-33.049
	HEMA	-30.090	-11.089	-19.128	-31.384	-37.280	-10.800	-26.997	-38.910
	NOBE	-36.003	-16.299	-20.615	-38.075	-41.165	-9.596	-31.590	-39.771

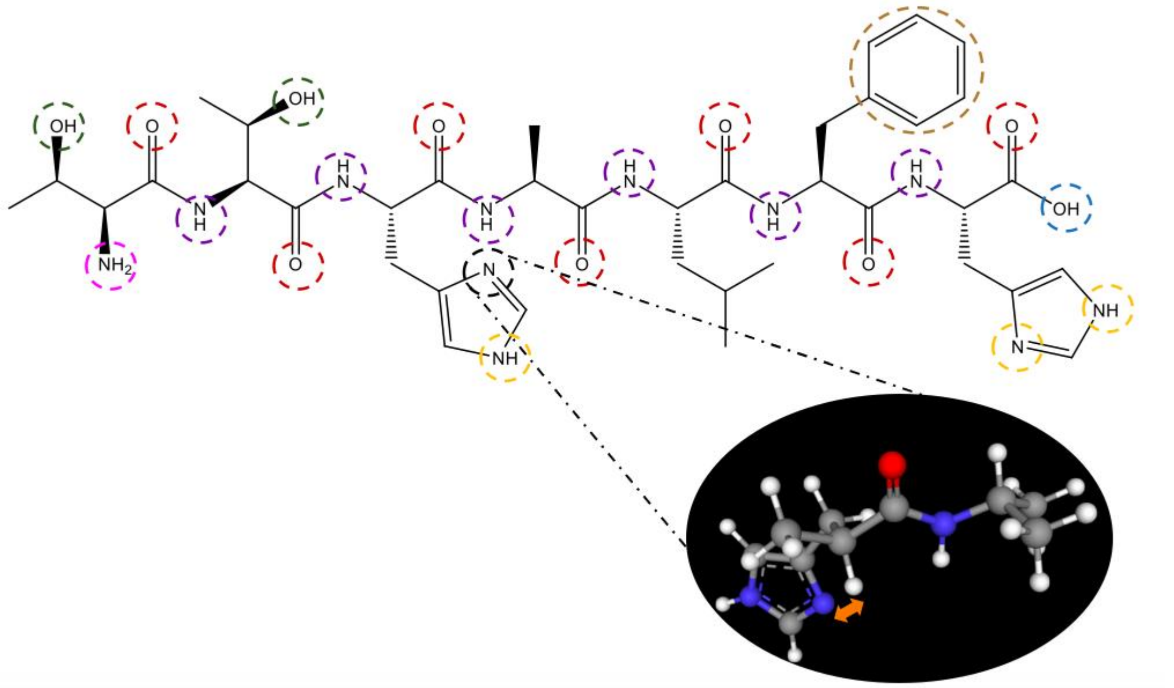

Figure S2. Structure of HAV epitope 2 (THR70-HIS76) with 21 active binding sites: 2 O atoms from hydroxyl groups (green), 1 O atom from carboxylic group (blue), 7 O atoms from six amide groups and one carboxyl group (red), 1 C atom from benzyl group (brown),6 N atoms from six amide groups (purple), 1 N atom from primary amine group (pink),4 N atoms from heterocyclic amine groups (yellow and black); however, one of them may not be available due to the steric hindrance.

Reagents and chemicals

Sodium hydroxide (NaOH), toluene anhydrous (99.8%), 25% aqueous glutaraldehyde (GA), sodium borohydride, ethanolamine, N-(3-aminopropyl) methacrylamide hydrochloride (APMA), 2-acrylamido-2-methylpropane sulfonic acid (AMPSA), 2-hydroxyethylmethacrylate (HEMA), 2-(methacryloyloxy)ethyl phosphate (MEP), ammonium persulfate (APS), N,N,N′,N′-tetramethylethylenediamine (TEMED), sodium borohydride ($NaBH_4$), phosphate buffered saline (PBS) tablets, 11-mercaptoundecanoic acid (MUDA), N-hydroxysuccinimide (NHS), sodium acetate, 2-(N-morpholino) ethanesulfonic acid (MES), TWEEN 20 (polyoxyethylenesorbitan monolaurate) were acquired from Sigma Aldrich (Taufkirchen, Germany). Glass beads with diameter of 0.75-1.0 mm, 25% ammonia, 1-ethyl-3-(3-dimethylaminopropyl) carbodiimide (EDC) were purchased from Carl Roth (Karlsruhe, Germany). (3-Aminopropyl)trimethoxysilane (APTMS) was purchased from Thermo Fisher Scientific (Germany) and methacryloxyethyl thiocarbamoyl rhodamine B was acquired from

Polysciences (USA). The PVDF membranes (Roti®-PVDF) were supplied by Carl Roth Corporation (Karlsruhe, Germany), provided in rolls measuring 375 × 26.5 cm with a pore size of 0.45 µm. Commercial PES membranes (Supor®-PES membranes) were purchased from Pall Corporation (New York, United States) in a format of 50 mm-diameter disks, featuring a pore size of 0.45 µm. Low-molecular-weight chitosan (deacetylated chitin, poly(D-glucosamine)) with a molecular weight ranging from 50,000–190,000 g mol^{-1} was purchased from Sigma-Aldrich Chemical Co. (Steinheim, Germany). Glacial acetic acid (ReagentPlus® glacial, ≥ 99%), and sodium hydroxide (NaOH, purity ≥98%) pellets were provided by Sigma-Aldrich Chemical Co. (Steinheim, Germany) and VWR International LLC. (Darmstadt, Germany), respectively. Ethanol (absolute 99.9%), acetonitrile (ACN; HPLC grade ≥ 99.9%), hydrochloric acid (HCl; 37-38% concentration), and phosphate-buffered saline (PBS) were also supplied by Sigma-Aldrich Chemical Co. (Steinheim, Germany). HAV particles, acquired as the inactivated, non-adsorbed hepatitis A vaccine, European Pharmacopoeia (Ph. Eur.) Biological Reference Preparation (BRP), with a concentration of 1350 IU mL^{-1}, was donated by researchers from the Free University of Berlin, Germany. Inactivated human adenovirus serotype 5 (AdV) and herpes simplex virus type 1 (HSV) were provided by researchers at the Free University of Berlin. Commercial human serum (Sigma Aldrich, Taufkirchen, Germany) was used for experiments performed with human serum samples.

Instrumentation

Hydrodynamic size and zeta potential measurements were performed with a Zetasizer Pro (Malvern Panalytical Ltd, Herrenberg, Germany). An FTIR spectrometer with a single reflection diamond attenuated total reflection (ATR) module in the range of 4000–650 cm^{-1} (Cary 630 FTIR, Agilent Technologies, Germany) was used to analyze chemo-functional groups present in freeze-dried CIRs. A confocal Raman spectroscope (Witec Alpha 300RA, Ulm, Germany), equipped with an argon (Ar) laser excitation line of 532 nm, a power of 5 mW in the range of 250–3750 cm^{-1} with spectral resolution 5 cm^{-1}, was used to investigate Raman spectral measurements of CIRs. Integration time was maintained for 25 s and spectra were recorded as the mean of three determinations. Both CIR polymerization mixtures contained rhodamine dye as a fluorescent dye-carrying monomer, however for Raman analysis, CIRs were synthesized in absence of the dye monomer to prevent fluorescent interference.

An X-ray photoelectron spectroscopy (XPS, XPS UHV system from PREVAC Sp. z o. o., Al-anode, 300W) was utilized to investigate the chemical composition of both CIR-1 and CIR-2. Scans were acquired at three iterations and a pass energy of 200 eV while high-resolution scans

were performed at 20 iterations and a pass energy of 50 eV. The software CasaXPS (version 2.3.23) was used to analyze XPS spectra. The Shirley algorithm was also applied to quantify the background of each spectrum. The charge correction was conducted by fitting the C 1s main peak and setting the peak position of the fit to 284.8 eV while adjusting all belonging spectra accordingly.

Fluorescence microscope BZ-X810 (Keyence, Germany) was used for visual characterization of sensor and membrane surfaces. QCM-I with impedance and dissipation measurements along with 5MHz QCM crystals (Microvacuum Ltd., Hungary) were utilized for mass—based detection assays. Affinity analysis of MIP particles was performed using Biacore X100 (Cytiva, Germany).

SCA 20 software, version 5.0, provided by DataPhysics (Filderstadt, Germany) was used for obtaining contact angle images at three different points by applying a water droplet onto their surfaces.The amplitude, phase distribution and surface topography of PVDF and PES membranes before and after chitosan modification and CIR immobilization, were analyzed using an atomic force microscopy (AFM; WITec alpha 300R, WITec GmbH, Ulm, Germany). This analysis was conducted in a contact mode at room temperature on 10 μm × 10 μm scanning area. Root mean square (RMS) roughness of images was calculated by using Gwyddion software.

The surface morphology of PVDF and PES membranes after CIR immobilization was analyzed using scanning electron microscopy (SEM; Gemini SEM Ultra55 Plus model, Zeiss, Germany). SEM micrographs were obtained at an acceleration voltage of 5 KV, with a magnification of 15,000 X, utilizing an InLens detector in order to achieve a good contrast, sharp outlines with any cracks in the gold layer. Before SEM analysis, samples were sputtered with gold layers (Vac Coat Desk Sputter and carbon Coater DSCR; I = 10 mA, time = 300 s) in an Ar plasma medium since membranes after functionalization are non-conductive polymers.

Synthesis of N,O-bismethacryloyl ethanolamine (NOBE)

N,O-bismethacryloyl ethanolamine (NOBE) monomer was synthesized following a previously reported procedure [1].

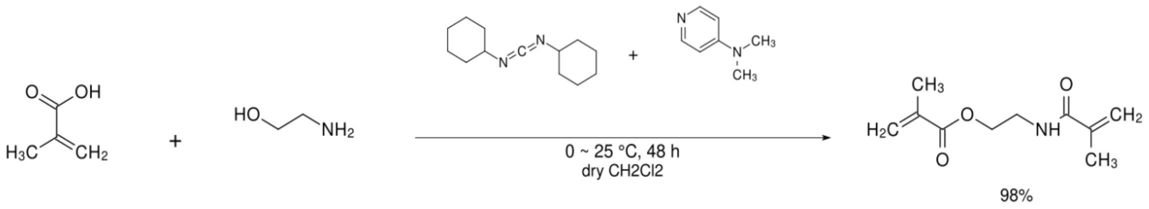

Figure S3. Synthesis procedure of NOBE monomer.

In a 500 mL round bottom flask, ethanolamine (4.019 g, 65.8 mmol) and 225 mL CH_2Cl_2 were cooled to 0°C. 4-Dimethylaminopyridine (1.637 g, 13.4 mmol) was added to the solution followed by the addition of methacrylic acid (12.66 g, 147 mmol). N,N'-dicyclohexylcarbodiimide (29.14 g, 141 mmol) was added after the solution equilibrated for 5 minutes. The mixture was slowly warmed to room temperature and stirred for 2 days. The DCU was filtered off by vacuum filtration and the organic solution was washed with 1 N HCl (aq) (3 x 200 mL) and sat. $NaHCO_3$ (aq) (3 x 200 mL.) The organic layer was dried over $MgSO_4$ and the solvent evaporated to obtain an oil with 98% yield. Resulting NMR analysis was given in Figure S2.

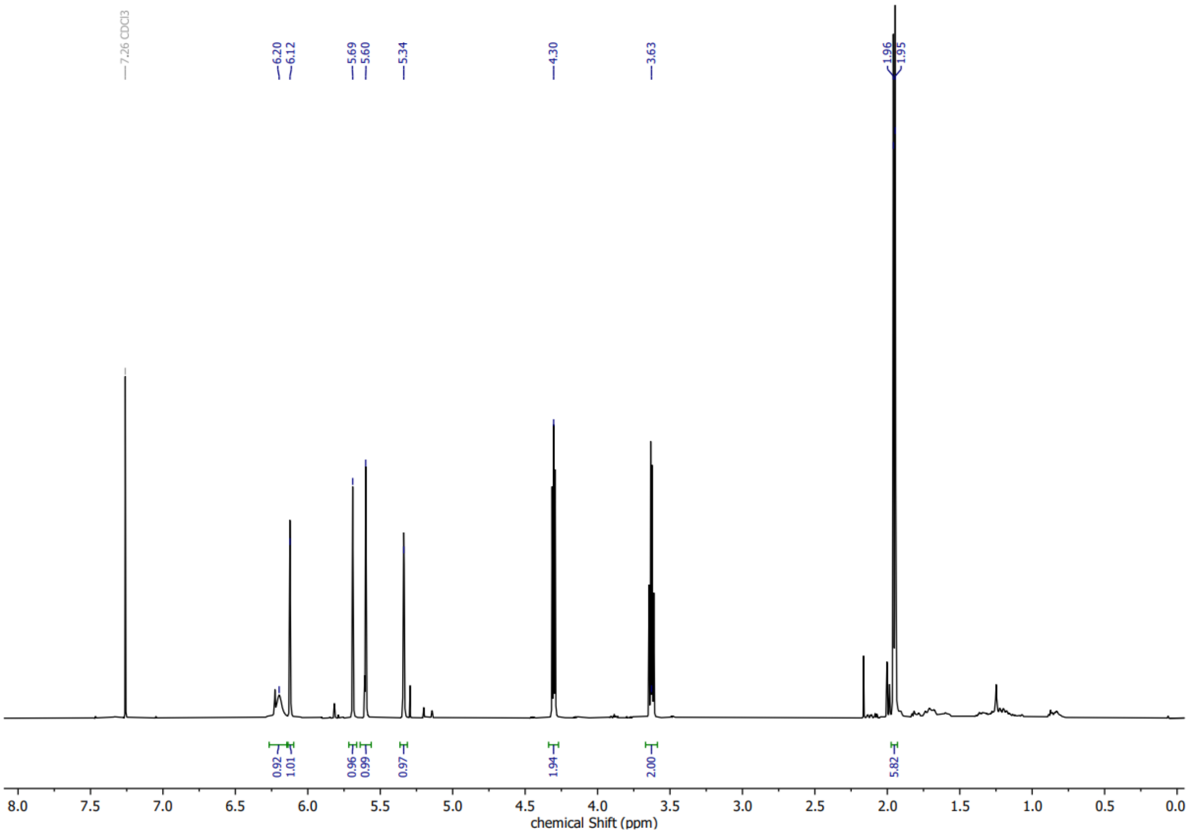

Figure S4. NMR analysis of in-house synthesized NOBE monomer.

NOBE: ^{1}H NMR (500 MHz, 298 K, $CDCl_3$) δ = 6.20 (1H, br, NH), 6.13-6.10 (dq, J= 1.5 Hz, 1 Hz, 1H, acrylate ester H trans to CH_3), 5.69 (p, J= 1 Hz, 1H acrylate ester H cis to CH_3), 5.60 (p, J= 1.6 Hz, 1H, acrylate amide H trans to CH_3), 5.34 (qd J= 1.6 Hz, 1 Hz, 1H, acrylate amide H cis to CH_3), 4.30 (t, J= 5.4 Hz, 2H), 3.63 (q, J= 5.4 Hz, 2H), 1.96 (dd, J= 1.5 Hz, 1 Hz, 3H, CH_3 from ester), 1.94 (J= 1.6 Hz, 1 Hz, 3H, CH_3 from amide) ppm.

Synthesis of 2,6-bis(acrylamido) pyridine (BAP)

A previously reported procedure with modifications was used for synthesis of 2,6-bis(acrylamido) pyridine (BAP) [2].

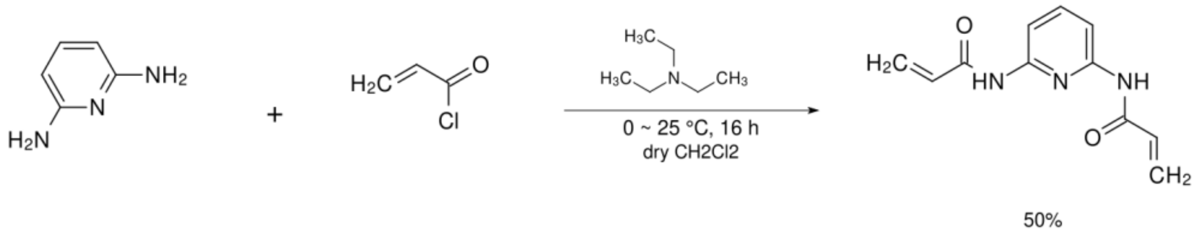

Figure S5. Synthesis procedure of BAP monomer.

To a solution of 2,6-diaminopyridine (10.0 g, 91.63 mmol), triethylamine (29.2 mL, 210.76 mmol), and CH_2Cl_2 (750 mL) was added acryloyl chloride (17.8 mL, 210.76 mmol) slowly over 20 min at 0°C. After the addition was completed, the mixture was allowed to warm up to room temperature while stirring overnight. Sat. $NaHCO_3$ -Solution (50 mL) was added to quench the remaining acryloyl chloride. The mixture was washed with sat. NaHCO3-Solution (4x75 mL) and with brine (1x100 mL). The organic layer was collected and dried over $MgSO_4$, and the residue was purified by column chromatography (Cy/EA 30-60). The isolated yellow solid was dissolved in the smallest possible amount of CH_2Cl_2 and subsequently precipitated in pentane. Percentage yield was calculated as 50%. The obtained solid was further investigated with NMR spectroscopy (Figure S4).

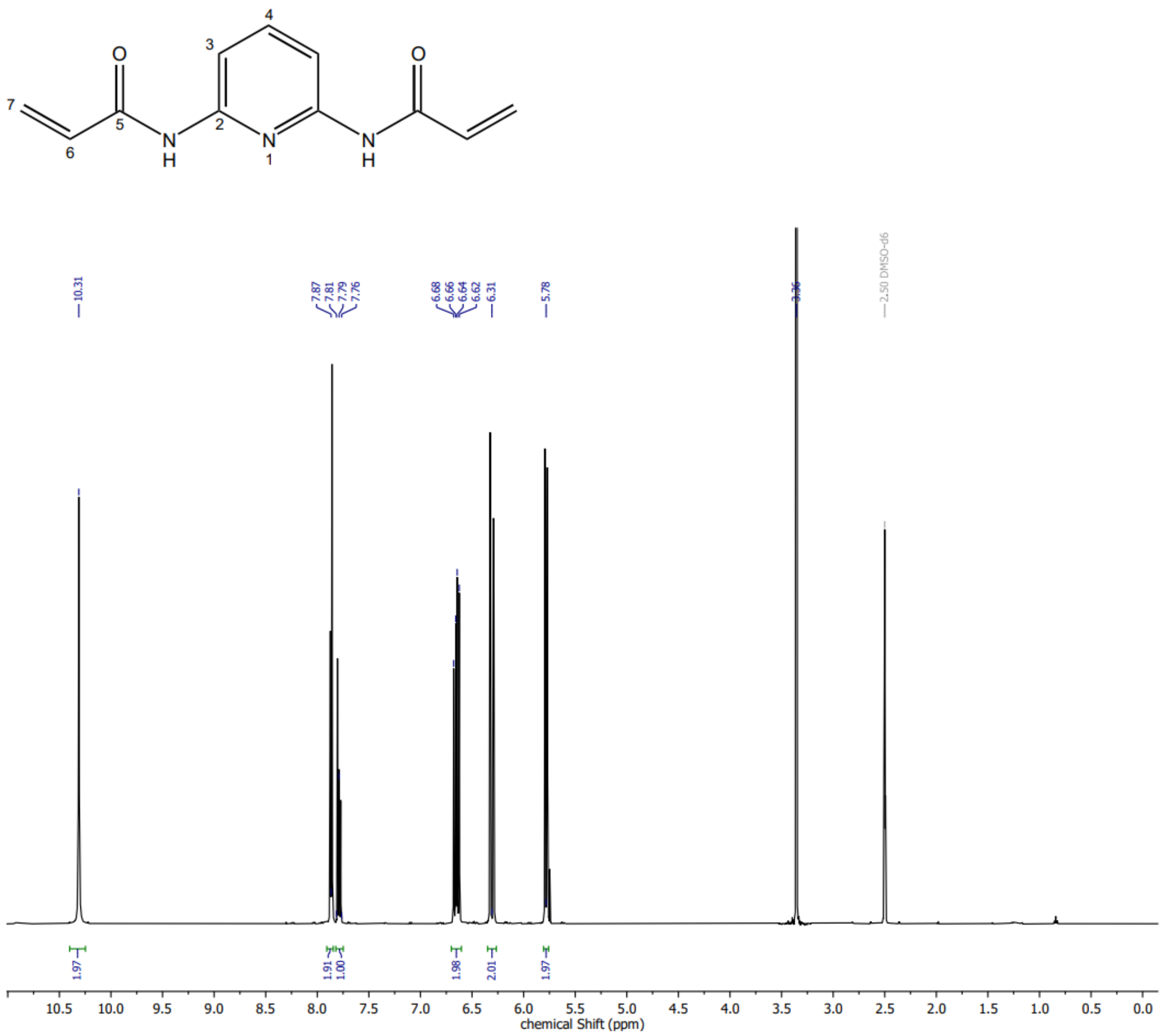

Figure S6. Resulting NMR spectrum of in-house synthesized BAP monomer.

BAP: ^{1}H-NMR (500 MHz, 298 K, DMSO-d6): δ = 10.31 (s, 2H, NH), 7.86 (dd, J = 1.3 Hz, 7.5 Hz, 2H, H-3), 7.81-7.76 (m, 1H, H-4), 6.65 (dd, J = 10.1 Hz, 16.9 Hz, 2H, H-6), 6.31 (dd, J = 2.0 Hz, 16.9 Hz, 2H, H-7 trans), 5.78 (dd, J = 2.0 Hz, 10.1 Hz, 2H, H-7 cis) ppm.

.

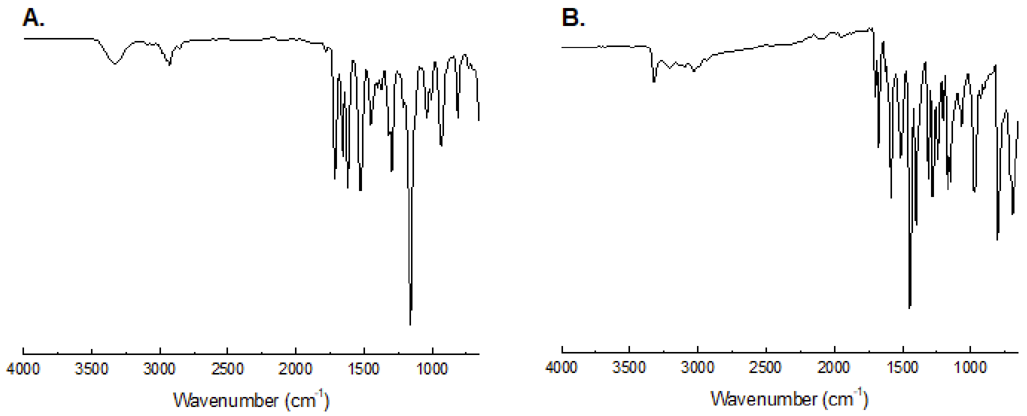

Figure S7. FTIR spectra of NOBE (A) and BAP (B) monomers.

Characterization of monomers

Further characterization of the monomers was performed with Fourier transform infrared spectroscopy (FTIR) measurements. FTIR spectra revealed peaks at a higher wavenumber region represent N-H stretching from amide groups and the C-H stretching in the molecular structure were observed for both functional monomers [3]. Furthermore, the C=O stretching peak characteristic of primary amide groups and carboxylates was observed around 1700 cm^{-1} with free associated peak at around 1640 cm^{-1} coming from secondary amide [4]. C=C stretching peaks at 1520 cm^{-1} were observed for both monomers [5]. A characteristic peak, C-N stretching, was observed at 1450 cm^{-1}, and it was more pronounced for BAP spectrum since it has three times more C-N bonds [6]. Peaks at 1310 cm^{-1} (N-H bending), 1170 cm^{-1} (C-O stretching), 1065 cm^{-1} (C=C bending), 975 cm^{-1} (C=C stretching from vinyl groups), 800 cm^{-1} (C-H stretching) and 695 cm^{-1} (C-H bending) were observed in BAP spectrum [6]. Peaks at 1310 cm^{-1} (N-H bending), 1170 cm^{-1} (C-O stretching), 1065 cm^{-1} (C=C bending), 940 cm^{-1} (C=C stretching from vinyl groups), 820 cm^{-1} (C-H stretching) and 655 cm^{-1} (C-H bending) were observed in NOBE spectrum [7].

Synthesis of Hepatitis A virus epitope

The epitope (TTHALFH) selected from viral protein 2 of HAV capsid was synthesized as follows:

Loading of the Resin: 2-CTC resin (5 g, 1.6 mmol/g) was pre-swollen for 10 min in DCM (30 ml) in a solid phase peptide synthesis vessel. After the solvent was drained, Fmoc-His(Trt)-OH (3.0 mmol) and DIPEA (10 mmol) in DCM (20 mL) were added to the resin. The mixture

was agitated for 2 h and then the solvent was drained. The resin was washed with DCM (3 x 20 mL). Then a mixture of MeOH/DIPEA/DCM (1:1:8, 20 ml) was added to cap the remaining 2-chlorotrityl chloride bound to the resin. The mixture was agitated for 30 min. Then the solvent was drained and the resin was washed with DMF (4 x 20 ml).

Removal of the Fmoc group: A solution of 20% piperidine in DMF (15 mL) was added to the resin and the resulting suspension was agitated for 10 min. Then the solution was drained and the step was repeated. The solution was drained and the resin was washed with DMF (5 x 20 mL).

Amino acid coupling: The next Fmoc-protected amino acid according to the amino acid sequence (Fmoc-Phe-OH, Fmoc-Leu-OH, Fmoc-Ala-OH, Fmoc-His(Trt)-OH, Fmoc-Thr(*t*Bu)-OH, Fmoc-Thr(*t*Bu)-OH) (2.0 eq) and HATU (1.9 eq) were dissolved in DMF (20 mL). DIPEA (12 eq) was added dropwise to the DMF solution. After activating for 1 min, the resulting solution was added to the Fmoc-deprotected resin-bound amino acid/peptide. The mixture was agitated for 30 min. Then, the solution was drained and the resin was washed with DMF (4 x 20 mL).

Cleavage from the resin*:* A mixture of TFA/TIS/H_2O (95:2.5:2.5; 50 ml) was added to the resin and agitated for 1 h at room temperature. The resin was filtered and washed with TFA/TIS/H2O (95:2.5:2.5; 20 ml). The filtrate was added dropwise to diethyl ether at 0°C. The resulting precipitate was centrifuged and the solution was then decanted off. The precipitate was washed three times with cooled diethyl ether. After lyophilization, the peptide was obtained as a white solid (2.2 g, 2.7 mmol, 89%).

High resolution mass spectroscopy (HRMS) and NMR measurements were performed for chemical analysis of the synthesized epitope (Figure S8).

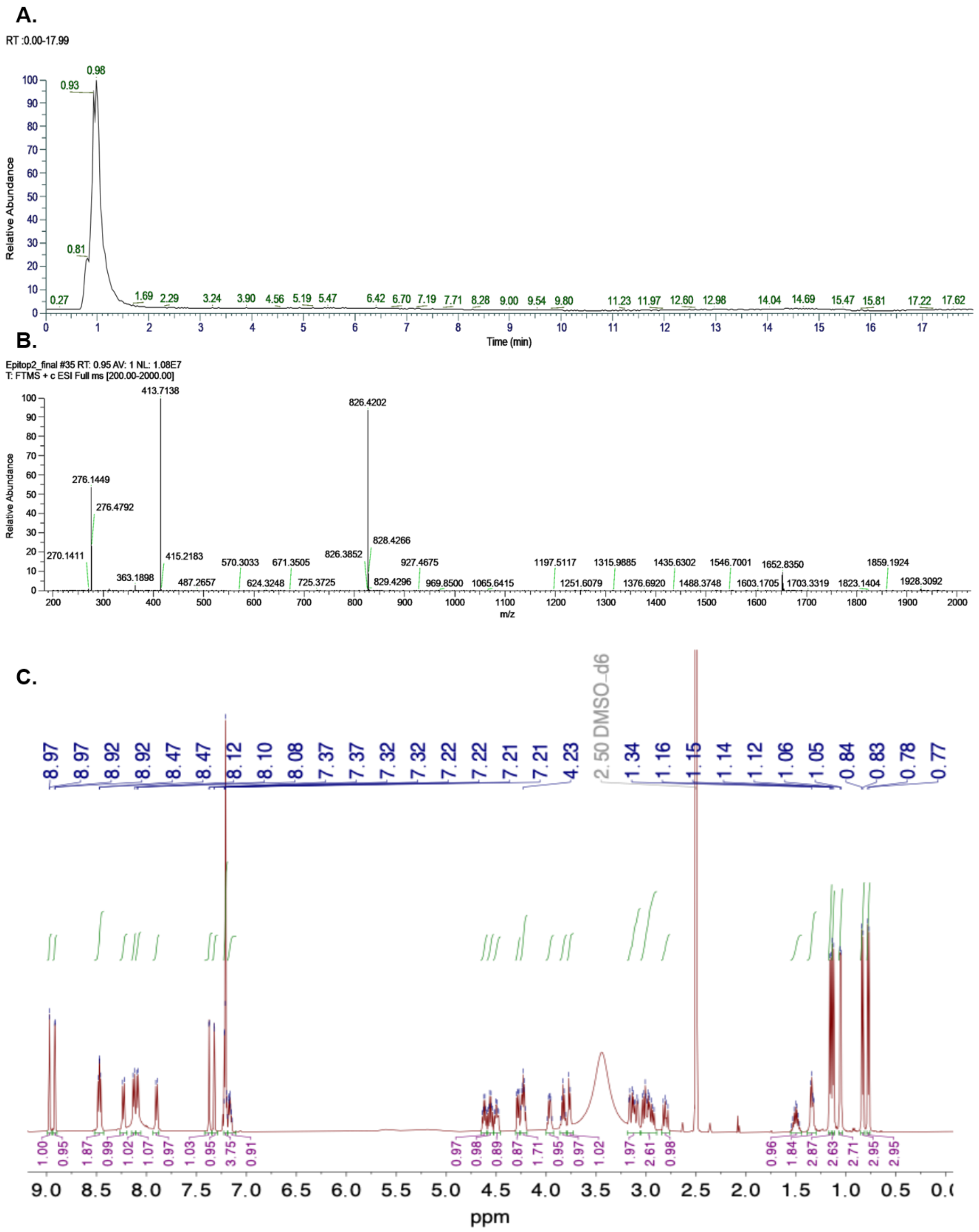

Figure S8. HRMS (A and B) and NMR (C) characterization of in-house synthesized HAV epitope 2.

^{1}H-NMR (500 MHz, DMSO) δ(ppm) = 8.97 (d, *J* = 1.4 Hz, 1H), 8.92 (d, *J* = 1.4 Hz, 1H), 8.47 (dd, *J* = 8.0, 6.1 Hz, 2H), 8.23 (d, *J* = 8.1 Hz, 1H), 8.13 (d, *J* = 6.7 Hz, 1H), 8.09 (d, *J* = 8.2 Hz, 1H), 7.90 (d, *J* = 8.0 Hz, 1H), 7.37 (d, *J* = 1.3 Hz, 1H), 7.32 (d, *J* = 1.3 Hz, 1H), 7.23 – 7.19 (m, 4H), 7.19 – 7.10 (m, 1H), 4.62 (td, *J* = 8.0, 5.4 Hz, 1H), 4.56 (td, *J* = 8.3, 5.6 Hz, 1H), 4.50

(td, J = 8.6, 4.5 Hz, 1H), 4.28 (dd, J = 8.0, 4.4 Hz, 1H), 4.23 (tt, J = 8.6, 5.0 Hz, 2H), 4.00 – 3.93 (m, 1H), 3.83 (p, J = 6.3 Hz, 1H), 3.77 (d, J = 6.8 Hz, 1H), 3.19 – 3.06 (m, 2H), 3.05 – 2.89 (m, 3H), 2.81 (dd, J = 14.1, 9.3 Hz, 1H), 1.50 (dp, J = 13.4, 6.7 Hz, 1H), 1.39 – 1.30 (m, 2H), 1.16 (d, J = 7.1 Hz, 3H), 1.13 (d, J = 6.3 Hz, 3H), 1.05 (d, J = 6.3 Hz, 3H), 0.83 (d, J = 6.6 Hz, 3H), 0.78 (d, J = 6.5 Hz, 3H).

Ninhydrin test

To verify whether the epitope successfully binds to the glass beads, a ninhydrin test was conducted before and after epitope incubation during the solid phase synthesis. Small aliquots of the solution were taken both before adding the epitope to the beads and after incubation. These samples were then mixed with 2% ninhydrin in ethanol and heated in a water bath at 80°C for 10 minutes [8]. The pre-incubation sample, taken directly from the stock epitope solution, reacted with ninhydrin, producing a purple color (Figure S9). This color change was due to the presence of the N-terminal threonine (THR70), which has a free amine group available for reaction. However, after epitope incubation with the glass beads, the ninhydrin test showed no visible color change (Figure S9). This result aligns with expectations, as it suggests that the epitope successfully bound to the glass beads.

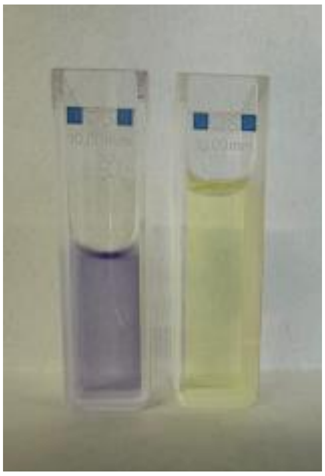

Figure S9. Colors of ninhydrin test before (left) and after (right) epitope incubation.

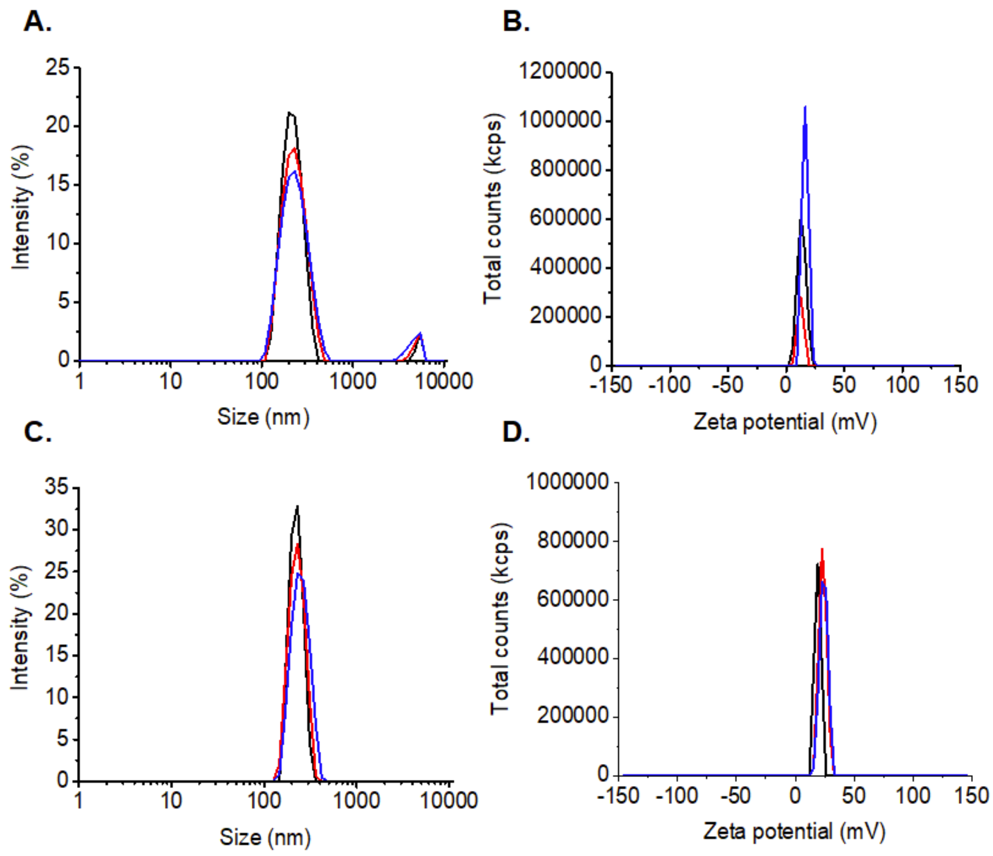

Figure S10. Hydrodynamic size and zeta potential of (A, B) CIR-1 and (C, D) CIR-2, respectively.

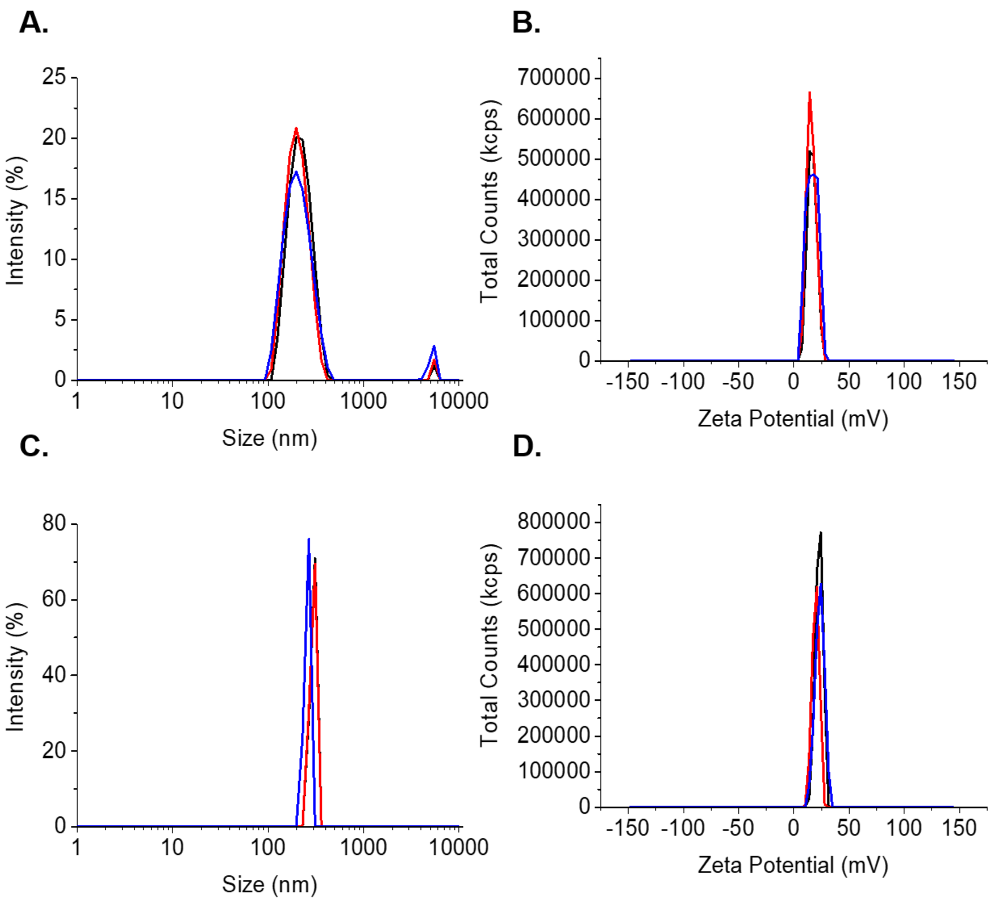

Figure S11. Hydrodynamic size and zeta potential of (A, B) NIP-1 and (C, D) NIP-2 respectively.

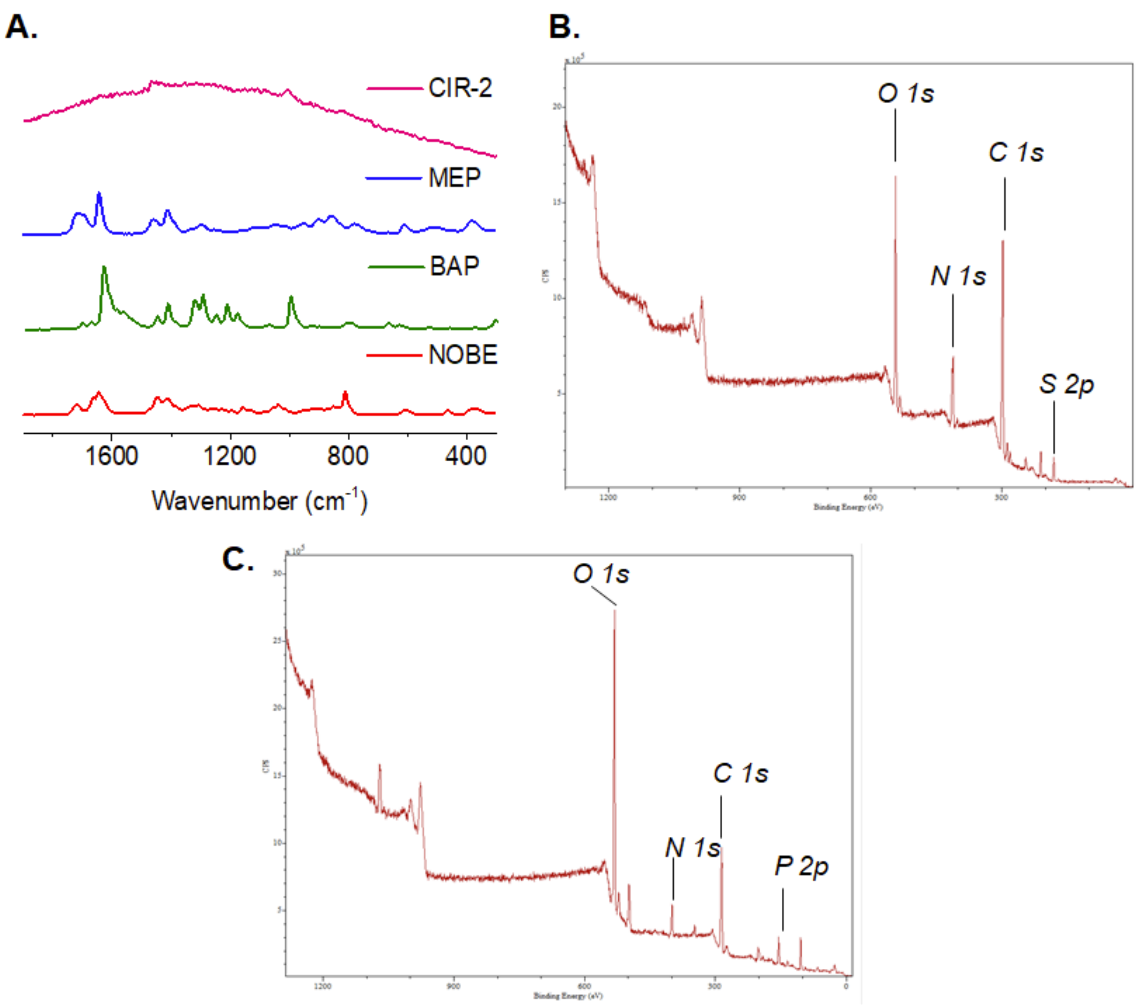

Figure S12. (A) Raman spectrum of CIR-2 together with its functional monomers. XPS white spectra of (B) CIR-1 and (C) CIR-2.

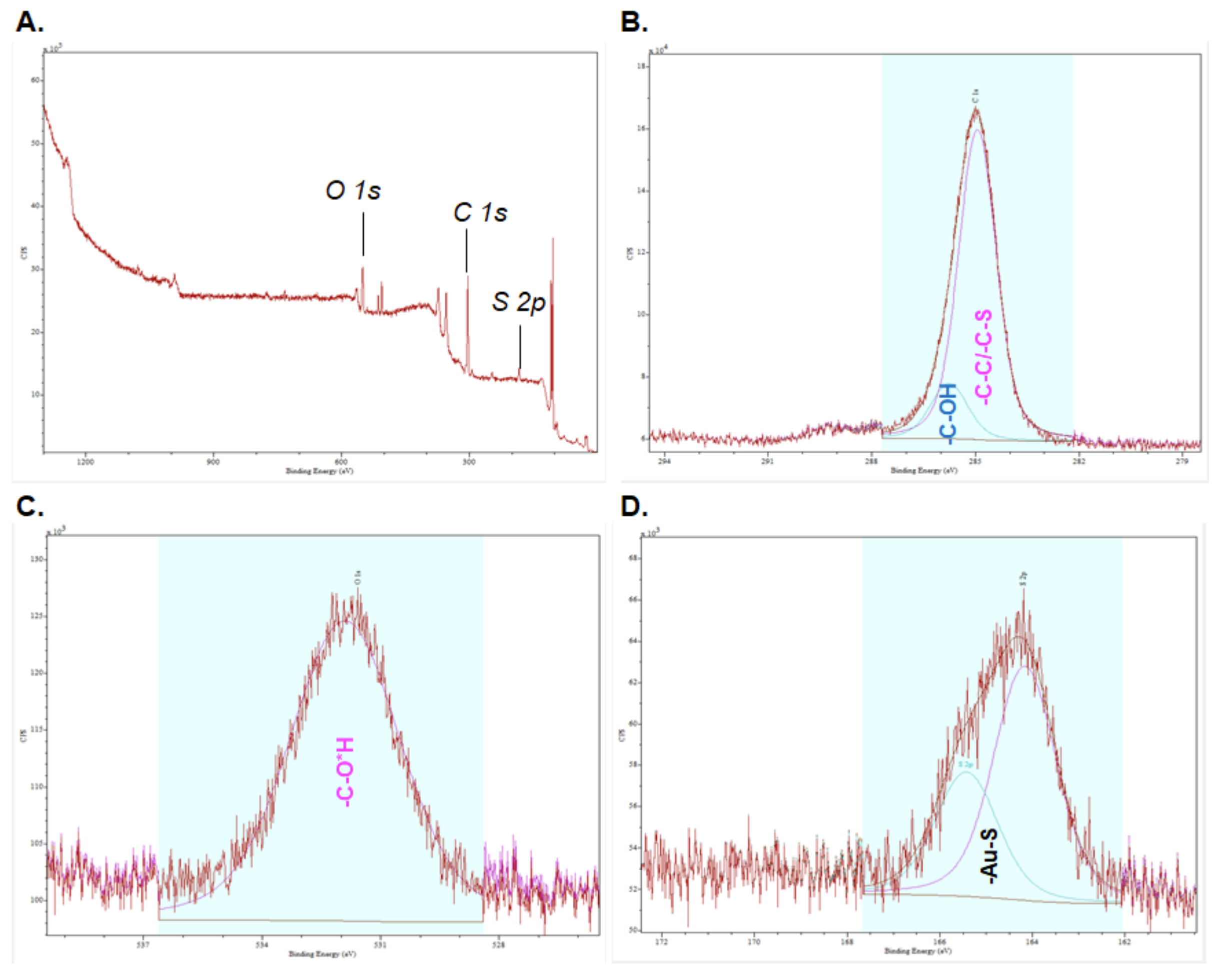

Figure S13. XPS results for MUDA coated gold QCM substrate with (A) white (B) carbon, (C) oxygen, and (D) nitrogen spectra.

Characterization of CIRs

The synthesized CIRs were characterized by FTIR to confirm the successful synthesis (Figure 5A). Peaks from both CIRs at wavenumber region between 3500 cm^{-1} and 2250 cm^{-1} representing N-H stretching from primary and secondary amine groups in the polymer combining with hydroxyl group stretching from intramolecular hydrogen bonding, were identified [9]. Furthermore, the C=O stretching peak characteristic of primary amide groups and carboxylates was observed around 1720 cm^{-1} in CIR-1 with a characteristic peak, N-H bending at 1650 cm^{-1} [10]. In contrast, CIR-2 showed broader C=C peak at 1640 cm^{-1}. Peaks at 1445-1395 cm^{-1} and 1040 cm^{-1} were attributed to the stretching of N-O and C-O in the polymer composition [11]. Furthermore, S=O stretching from AMPSA monomer in CIR-1 contributed to the C-O stretching peak [12]. Also, C=C and C-H bending peaks appeared around 795 cm^{-1} [13]. Overall, the intensity and broader peaks at high wavenumber region of CIR-2 indicated a more hydrophobic nature in comparison to CIR-1 [5].

Raman spectroscopy was employed to identify functional groups in CIR-1 and CIR-2 by analyzing vibrational modes associated with specific molecular bonds, while also investigating the functional monomers to determine the origins of each peak. Figures 5B and S12A shows Raman spectra of CIR-1 and CIR-2, respectively, together with the individual monomers used for the synthesis of each ligand. The CIR-1 exhibits unique vibrational bands in fingerprint region, between 300 cm^{-1} and 1900 cm^{-1}. Vibrational frequencies located at 600 cm^{-1} (C-C-O stretching), 750 cm^{-1} (C-C-C stretching combined with C-H bending), 910 cm^{-1} ($C(CH)_3$), 1000 cm^{-1} (C-C stretching), 1105 cm^{-1} (CH_3) rocking, 1175 cm^{-1} (sulfonic acid functional group of AMPSA), 1440 cm^{-1} $(CH_2)_3$ bending, and 1580 cm^{-1} aromatic ring [14] . However, only vibrational frequencies located at 1000 cm^{-1} (C-C stretching) and 1450 cm^{-1} $(CH_2)_3$ bending were observed for CIR-2. There are two main reasons why Raman spectrum might not be observed: (i) fluorescence interference: fluorescence from the sample can overwhelm the Raman signal making it difficult to detect [15], and (ii) absence of Raman-active modes: some materials may not exhibit Raman scattering due to their molecular or crystal structure [16]. All functional monomers used in synthesis of CIR-1 and CIR-2 were investigated in Raman analysis in order to address this problem. The CIR-2 polymer showed a different Raman spectrum, although the monomers did not show neither any fluorescence interference nor Raman-inactive modes, since CIR-2 has different molecular structure and composition, stereoregularity, crystallization and orientation, molecular interaction, surface and interface structure than individual monomers (Figure S12A) [17]. Similar behavior was observed in FTIR spectrum of CIR-2 since both FTIR and Raman spectroscopy give information about vibration of molecules (atoms) and stretching of molecules (atoms) or lattice vibration.

X-ray photoelectron spectroscopy (XPS) analysis of CIR-1 (Figure 5C-F) and CIR-2 (Figure 5G-I) was performed to investigate the characteristic electron binding energies of their functional groups. For this analysis, polymeric ligands were covalently immobilized on 11-Mercaptoundecanoic acid (MUDA) modified QCM substrates using dimethylaminopropyl) carbodiimide (EDC) and N-hydroxysuccinimide (NHS) coupling chemistry. The obtained white spectra for CIR-1 and CIR-2 are given in Figure S12B-C. After obtaining white spectra for each sample, analysis of individual elements was performed. Both CIR-1 and CIR-2 functionalized samples exhibited C 1s peaks at ~285.5 eV (-C=C/-C=H/-C-C/-C-H), ~286.5 eV (-C-N/-C-O), and ~288.8 eV (-O-C=O) [18,19]. The presence of nitrogen is also confirmed by N 1s peaks for pyridinic nitrogen and -NH-C=O [18,19]. The CIR-1 high resolution XPS spectrum of oxygen (O 1s) deconvolved into two peaks with binding energies ~528 eV and ~530 eV corresponding to $–SO_3H$ and -O-H/-O-C=O/-N-C=O respectively, while CIR-2 high resolution

spectrum displays ~529.5 eV (-O=P) and ~531 eV (-O-C/-O-P/-O-H/-O-C=O/-N-C=O). AMPSA functional monomer displays characteristic sulphur (S 2p) peak at ~166.5 eV, indicating the presence of sulphur groups on the CIR-1 surface [20]. Moreover, MUDA coated substrate was investigated as a reference, confirming the presence of carbon C 1s peaks for -C-OH and –C-C/-C-S, while oxygen (O 1s) and nitrogen (N 1s) peaks reflect –C-OH and –Au-S functional group contributions (Figure S13) [21]. XPS characterization confirmed that both CIR-1 and CIR-2 were successfully synthesized.

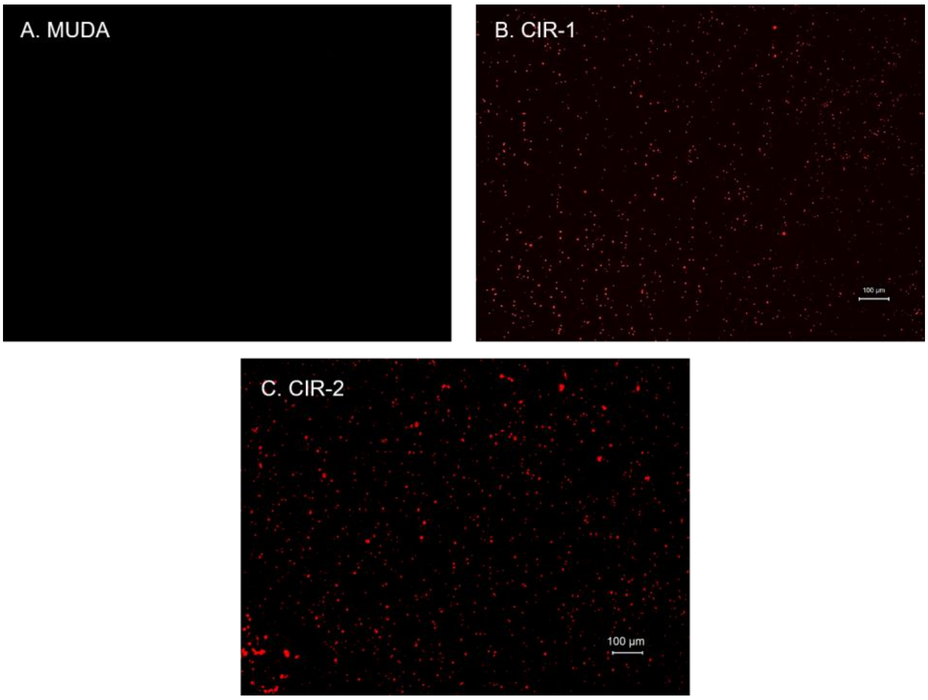

Figure S14. Fluorescence micrographs for MUDA, CIR-1 and CIR-2 functionalized QCM substrates.

Virus concentration conversion

HAV particles were acquired as the inactivated, non-adsorbed hepatitis A vaccine, European Pharmacopoeia (Ph. Eur.) Biological Reference Preparation (BRP), with a concentration of 1350 IU mL^{-1}, from researchers in the Free University of Berlin, Germany.[9] We converted this concentration to molar unit by using QCM as an ultrasensitive balance under certain assumptions. Firstly, we assumed that all of the injected virus particles are adsorbed on chip surface. Secondly, we assume that the HAV particles have spherical shapes with a diameter of 27 nm and a density of 1.32 g cm^{-22}. The experimental procedure went as follows:

A fresh QCM sensor chip was boiled in a cleaning solution containing 2 mL ammonia (25%), 2 mL hydrogen peroxide (35%) and 10 mL ultrapure water (UPW) for 10 minutes. The chip was thoroughly washed with UPW and dried with pressured air. The clean ship was incubated in 2 mM MUDA in ethanol overnight to obtain carboxylic acid groups on gold substrate. Next day, the sensor chip was assembled in QCM-I measurement chamber and PBS buffer was run through the system at the rate of 15 μL min^{-1}. The carboxylic acid groups were activated with a mixture of 0.2 M EDC and 0.05 M NHS for 4 minutes at 15 μL $min^{-1}$and 1 mL of 13.50 IU mL^{-1} in 10 mM sodium acetate buffer (pH 5) was injected into chamber. After completing the injection of virus solution, the chamber was washed with PBS. The stabilized frequency values recorded for PBS injection before and after virus adsorption were subtracted to calculate the frequency change ΔF. This measurement was repeated 3 times and average ΔF value was calculated as 37.56 Hz.

In order to convert the frequency change into mass adsorbed per area, Sauerbrey equation (Equation S1) was used[23]. Here, Δm refers to mass adsorbed per unit area, C is the mass-sensitivity of the crystal (17.7 ng cm^{-2} Hz^{-1} for the crystal used), n is the number of harmonics in which change in the resonance frequency (ΔF_n) recorded. Following this relation, total mass adsorbed on crystal was calculated as 521.9 ng.

$$\Delta m = -\frac{C}{n}\Delta F_n$$

Equation S1. Sauerbrey equation showing the effect of adsorbed mass on the resonance frequency change of piezoelectric crystal.

Assuming the spherical volume of HAV particle, the mass of one HAV particle was estimated as 1.36 x 10^{-17} g. Dividing the total adsorbed mass of HAV to the mass of one HAV particle, we calculated the number of adsorbed HAV as 383.73 x 10^{8} particles in 1 mL sample which corresponds to 64 pM in molar units.

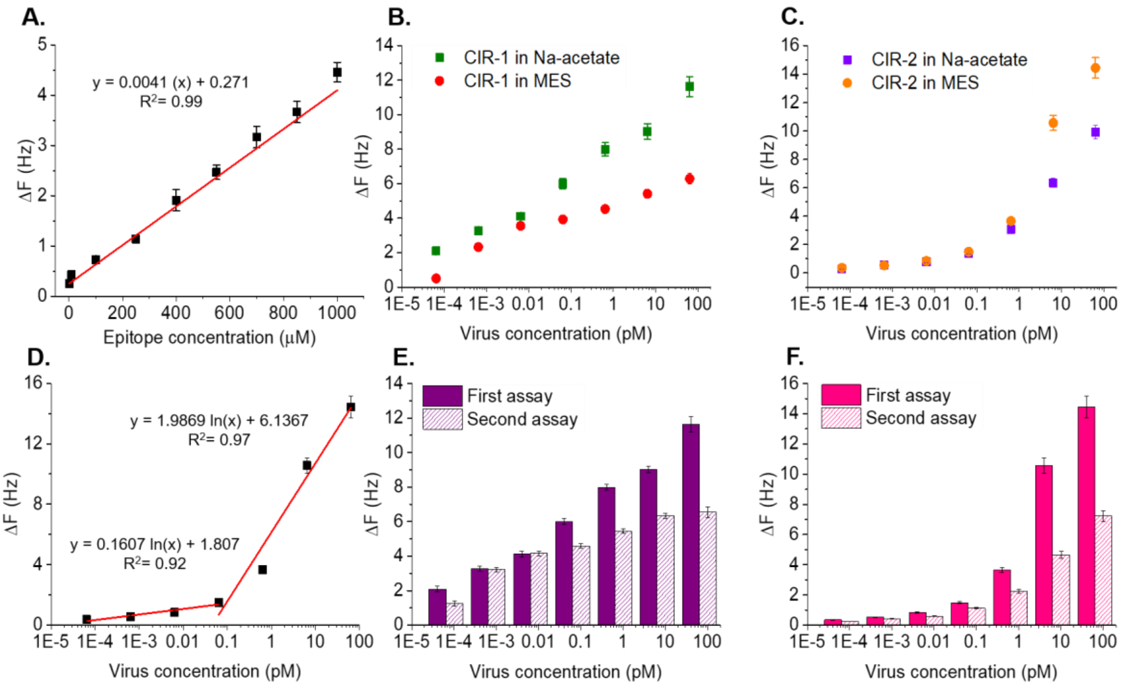

Figure S15. Concentration dependent frequency change of CIR-2 conjugated QCM crystal for HAV epitope range of 1 - 1000 µM (A). CIR-1 (B) and CIR-2 (C) HAV sensing response when two different immobilization buffers were utilized for ligand conjugation. Frequency change of CIR-2 conjugated QCM sensor for HAV concentration range of 0.064 fM - 64 pM with logarithmic regression analysis (D). HAV detection performance of CIR-1 (E) and CIR-2 (F) conjugated sensing platforms before and after regeneration with basic treatment.

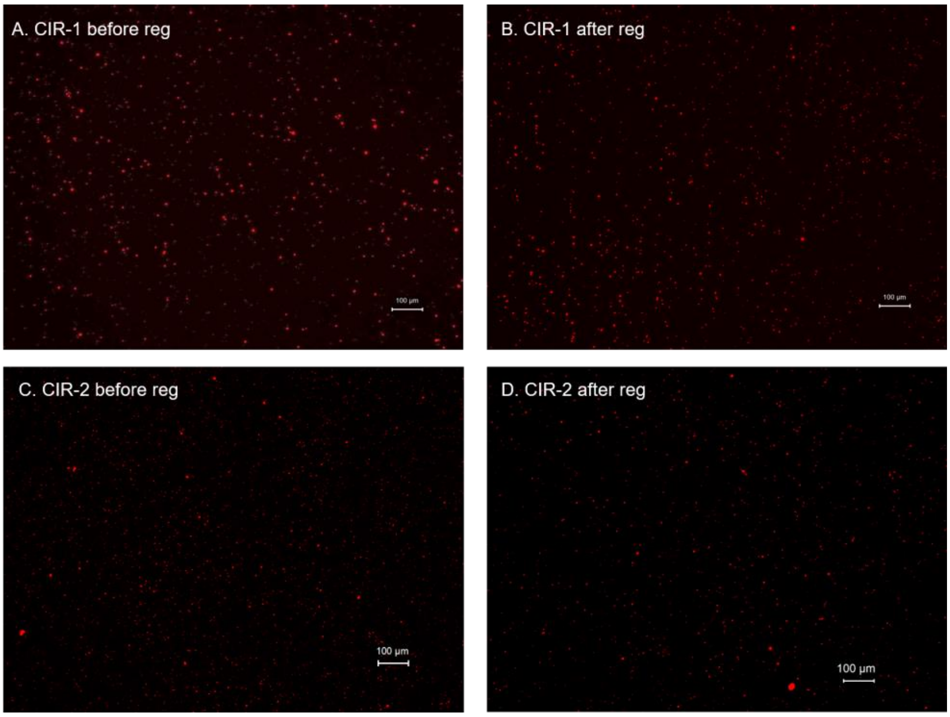

Figure S16. Fluorescence micrographs of CIR-1 and CIR-2 functionalized QCM substrates before and after basic treatment for regeneration.

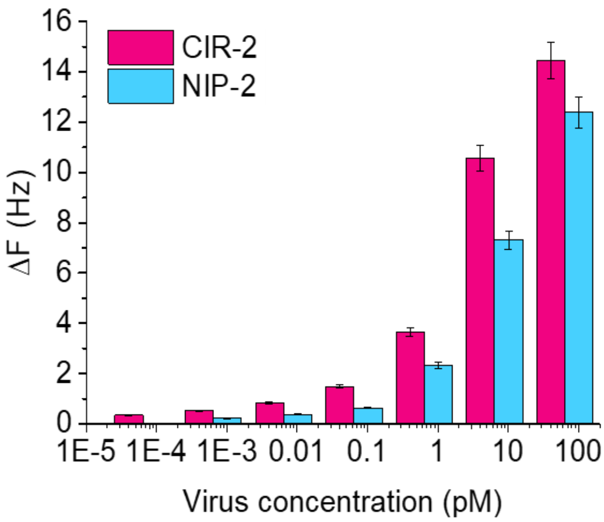

Figure S17. HAV detection with CIR-2 and control non-imprinted polymer (NIP-2) synthesized without epitope template.

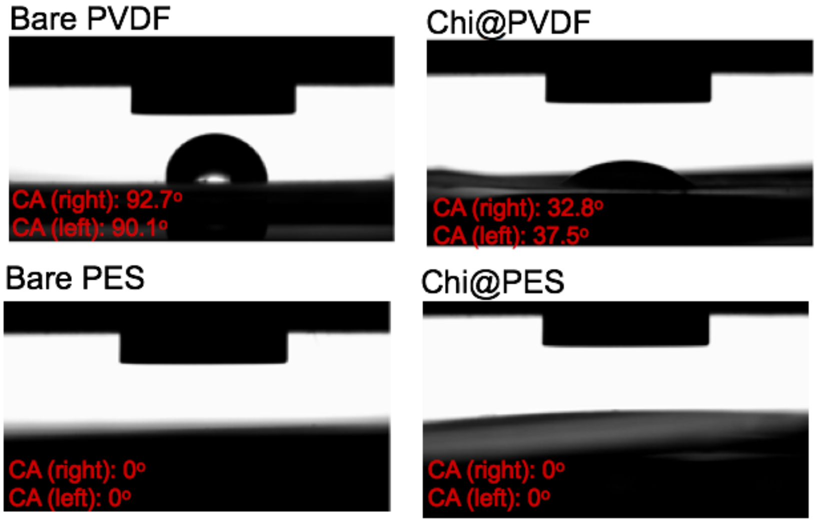

Figure S18. Goniometer images for contact angles (CAs) of bare PVDF and PES membranes, Chi@PVDF and Chi@PES.

Table S2. Coefficient of variation (CV) analysis across CIR-1 vs CIR-2 functionalized PVDF and PES membranes.

Membrane Type	W_dry_CV	W_wet_CV	DS_CV
Bare PVDF	0.014939	0.020767	0.033333
CF-PVDF	0.024534	0.012787	0.023256
CF-PVDF/CIR-1	0.017514	0.008002	0.020833
CF-PVDF/CIR-2	0.008466	0.007468	0.022727
Bare PES	0.018329	0.014624	0.021739
CF-PES	0.026938	0.009117	0.035714
CF-PES/CIR-1	0.015695	0.012260	0.016949
CF-PES/CIR-2	0.012450	0.008039	0.036364

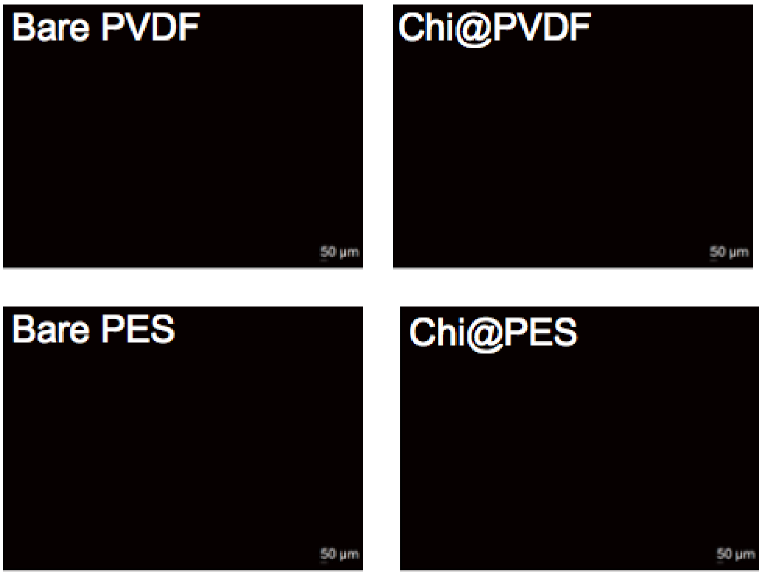

Figure S19. Fluorescence microscopy images of bare PVDF and PES membranes, Chi@PVDF and Chi@PES.

References

(1) Meador, D. S.; Spivak, D. A. Absolute Configuration Determination Using Enantiomeric Pairs of Molecularly Imprinted Polymers. *Org. Lett.* **2014**, *16*, 1402–1405.

(2) Duffy, D. J.; Das, K.; Hsu, S. L.; Penelle, J.; Rotello, V. M.; Stidham, H. D. Binding Efficiency and Transport Properties of Molecularly Imprinted Polymer Thin Films. *J. Am. Chem. Soc.* **2002**, *124*, 8290–8296. https://doi.org/https://doi.org/10.1021/ja0201146.

(3) Ishigaki, M.; Atsushi, I.; Hara, R.; Miyazaki, S.; Murayama, K.; Yoshikiyo, K.; Yamamoto, T.; Ozaki, Y. Method of Monitoring the Number of Amide Bonds in Peptides Using Near-Infrared Spectroscopy. *Anal. Chem.* **2021**, *93*, 2758–2766. https://doi.org/10.1021/acs.analchem.0c03424.

(4) Kar, A.; Karak, N. Bio-based Poly(Ester Amide): Mechanical , Thermal and Biodegradable Behaviors. *J. Polym. Res.* **2022**, *29*, 366. https://doi.org/10.1007/s10965-022-03214-z.

(5) Sadat, A.; Joye, I. J. Peak Fitting Applied to Fourier Transform Infrared and Raman Spectroscopic Analysis of Proteins. *Appl. Sci.* **2020**, *10*, 5918. https://doi.org/doi:10.3390/app10175918.

(6) Hasanah, A. N.; Safitri, N.; Zulfa, A.; Neli, N.; Rahayu, D. Factors Affecting Preparation of Molecularly Imprinted Polymer and Methods on Finding Template-Monomer Interaction as the Key of Selective Properties of the Materials. *Molecules* **2021**, *26*, 5612. https://doi.org/https://doi.org/ 10.3390/molecules26185612.

(7) Song, Z.; Li, J.; Lu, W.; Li, B.; Yang, G.; Bi, Y.; Arabi, M.; Wang, X.; Ma, J.; Chen, L. Molecularly Imprinted Polymers Based Materials and Their Applications in Chromatographic and Electrophoretic Separations. *TrAC Trends Anal. Chem.* **2022**, *146*, 116504. https://doi.org/https://doi.org/10.1016/j.trac.2021.116504.

(8) Stauß, A. C.; Fuchs, C.; Jansen, P.; Repert, S.; Alcock, K.; Ludewig, S.; Rozhon, W. The Ninhydrin Reaction Revisited : Optimisation and Application for Quantification of Free Amino Acids. *Molecules* **2024**, *29*, 3262. https://doi.org/https://doi.org/10.3390/molecules29143262.

(9) Akin, E.; Gharibzahedi, S. M. T.; Qiu, H.; Aliyeva, A.; Altintas, Z. Chitosan-Functionalized PVDF and PES Membranes Integrated by Epitope-Imprinted Polymers for Targeted Hepatitis A Virus Capture. *J. Memb. Sci.* **2024**, *709* (April), 123084. https://doi.org/10.1016/j.memsci.2024.123084.

(10) Gebrewbet, G. H.; Hndeya, A. G. Phytochemical Screening and Antibacterial Activity

Studies on the Crude Leaf Extract of Solanum Sisymbriifolium : Traditional Ethiopian Medicinal Plant. *Adv. Gut Microbiome Res.* **2023**, 5525606. https://doi.org/10.1155/2023/5525606.

(11) Dong, H.; Xiao, K.; Tang, X.; Zhang, Z.; Dai, J.; Long, R.; Liao, W. Preparation and Characterization of Polyurethane (PU)/Polyvinylidene Fluoride (PVDF) Blending Membrane. *Desalin. Water Treat.* **2016**, *57* (8), 3405–3413. https://doi.org/10.1080/19443994.2014.988659.

(12) Briggs, E.; Mensah, R. A.; Patel, K. D.; Mandakhbayar, N.; Sharifulden, N. S.; Erdogan, Z. K.; Silva, L. V. B.; Salim, K.; Kim, H.; Nguyen, L. T. B.; et al. Therapeutic Application of an Ag-Nanoparticle-PNIPAAm- Modified Eggshell Membrane Construct for Dermal Regeneration and Reconstruction. *Pharmaceutics* **2022**, *14,* 2162. https://doi.org/https://doi.org/10.3390/pharmaceutics14102162.

(13) Gola, A.; Sacharczuk, M.; Musiał, W. Synthesis of AMPSA Polymeric Derivatives Monitored by Electrical Conductivity and Evaluation of Thermosensitive Properties of Resulting Microspheres. *Molecules* **2019**, *24,* 1164. https://doi.org/10.3390/molecules24061164.

(14) Remanan, S.; Bose, M.; Das, A. K.; Das, N. C. Preparation and Characterization of a Unique Low-Cost Microfiltration Membrane from a Technologically Compatible Poly(Ethylene-Co-Methyl Acrylate)/Poly(Vinylidene Fluoride) Blend for Water Filtration Application. *J. Appl. Polym. Sci.* **2019**, *136* (12), 47218. https://doi.org/https://doi.org/10.1002/app.47218.

(15) Gelder, J. De; Gussem, K. De; Vandenabeele, P.; Moens, L. Reference Database of Raman Spectra of Biological Molecules. *J. Raman Spectrosc.* **2007**, *38,* 1133–1147. https://doi.org/10.1002/jrs.1734.

(16) Zhang, M.; Feng, Y.; Li, L.; Zhang, X.; Xu, F. Reducing Fluorescence Interference for Improved Raman Spectroscopic Analysis of Plant Cell Walls. *Wood Sci. Technol.* **2024**, *58,* 1697–1710. https://doi.org/https://doi.org/10.1007/s00226-024-01587-6.

(17) Pappas, P.; Liarokapis, E.; Calamiotou, M.; Bussman-Holder, A. Magnetic Interactions and the Puzzling Absence of Any Raman Mode in EuTiO3. *J. Raman Spectrosc.* **2021**, *52,* 914–924. https://doi.org/10.1002/jrs.6075.

(18) Sterner, E. S.; Rosol, Z. P.; Gross, E. M.; Gross, S. M. Thermal Analysis and Ionic Conductivity of Ionic Liquid Containing Composites with Different Crosslinkers. *J. Appl. Polym. Sci.* **2009**, *114* (5), 2963–2970. https://doi.org/https://doi.org/10.1002/app.30894.

(19) Fouda, A. S.; Khalil, E. M.; Mahdy, G. A. E. L.; Shaban, M. M.; Mohammed, A. S.; Abdelsatar, N. A. Synthesis and Characterization of Novel Acrylamide Derivatives and Their Use as Corrosion Inhibitors for Carbon Steel in Hydrochloric Acid Solution. *Sci. Rep.* **2023**, 1–16. https://doi.org/10.1038/s41598-023-30574-3.

(20) Castner, D. G.; Ratner, B. D.; Hirao, A.; Nakahama, S. Characterization of Poly(2-Hydroxyethyl Methacrylate) (PHEMA) by XPS. *Surf. Sci. Spectra* **1996**, *4* (1), 14–20. https://doi.org/10.1116/1.1247807.

(21) Li, W.; Wang, Q.; Cui, F.; Jiang, G. Covalent Organic Framework with Sulfonic Acid Functional Groups for Visible Light-Driven CO2 Reduction. *RSC Adv.* **2022**, *12*, 17984–17989. https://doi.org/10.1039/d2ra02660k.

(22) Mcknight, K. L.; Lemon, S. M. Hepatitis AVirus Genome Organization and Replication Strategy. *Cold Spring Harb. Perspect. Med.* **2018**, *8,* a033480. https://doi.org/10.1101/cshperspect.a033480.

(23) Sehit, E.; Yao, G.; Battocchio, G.; Radfar, R.; Trimpert, J.; Mroginski, M. A.; Süssmuth, R.; Altintas, Z. Computationally Designed Epitope-Mediated Imprinted Polymers versus Conventional Epitope Imprints for the Detection of Human Adenovirus in Water and Human Serum Samples. *ACS Sensors* **2024**, *9*, 1831–1841. https://doi.org/10.1021/acssensors.3c02374.
